# Next generation sequencing of triple negative breast cancer to find predictors for chemotherapy response

**DOI:** 10.1186/s13058-015-0642-8

**Published:** 2015-10-03

**Authors:** Esther H. Lips, Magali Michaut, Marlous Hoogstraat, Lennart Mulder, Nicolle JM Besselink, Marco J. Koudijs, Edwin Cuppen, Emile E. Voest, Rene Bernards, Petra M. Nederlof, Jelle Wesseling, Sjoerd Rodenhuis, Lodewyk FA Wessels, On behalf of the Center for Personalized Cancer Treatment

**Affiliations:** Department of Molecular Pathology, the Netherlands Cancer Institute, Plesmanlaan 121, 1066 CX Amsterdam, The Netherlands; Department of Pathology, Amsterdam, the Netherlands Cancer Institute, Plesmanlaan 121, 1066 CX Amsterdam, The Netherlands; Department of Molecular Carcinogenesis, the Netherlands Cancer Institute, Plesmanlaan 121, 1066CX Amsterdam, The Netherlands; Department of Medical Oncology, UMC Utrecht, Heidelberglaan 100, 3584 CX Utrecht, The Netherlands; Department of Medical Genetics, UMC Utrecht, Heidelberglaan 100, 3584 CX Utrecht, The Netherlands; Department of Medical Oncology, the Netherlands Cancer Institute, Plesmanlaan 121, 1066 CX Amsterdam, The Netherlands; Department of EEMCS, Delft University of Technology, Mekelweg 4, 2628 CD Delft, The Netherlands

## Abstract

**Introduction:**

In triple negative breast cancers (TNBC) the initial response to chemotherapy is often favorable, but relapse and chemotherapy resistance frequently occur in advanced disease. Hence there is an urgent need for targeted treatments in this breast cancer subtype. In the current study we deep sequenced DNA of tumors prior to chemotherapy to search for predictors of response or resistance.

**Methods:**

Next generation sequencing (NGS) was performed for 1,977 genes involved in tumorigenesis. DNA from 56 pre-treatment TNBC-biopsies was sequenced, as well as matched normal DNA. Following their tumor biopsy, patients started neoadjuvant chemotherapy with doxorubicin and cyclophosphamide. We studied associations between genetic alterations and three clinical variables: chemotherapy response, relapse-free survival and BRCA proficiency.

**Results:**

The mutations observed were diverse and few recurrent mutations were detected. Most mutations were in *TP53*, *TTN*, and *PIK3CA* (55 %, 14 %, and 9 %, respectively). The mutation rates were similar between responders and non-responders (average mutation rate 9 vs 8 mutations). No recurrent mutations were associated with chemotherapy response or relapse. Interestingly, *PIK3CA* mutations were exclusively observed in patients proficient for *BRCA1*. Samples with a relapse had a higher copy number alteration rate, and amplifications of *TTK* and *TP53BP2* were associated with a poor chemotherapy response.

**Conclusions:**

In this homogenous cohort of TNBCs few recurrent mutations were found. However, PIK3CA mutations were associated with BRCA proficiency, which can have clinical consequences in the near future.

**Electronic supplementary material:**

The online version of this article (doi:10.1186/s13058-015-0642-8) contains supplementary material, which is available to authorized users.

## Introduction

Neoadjuvant chemotherapy (NAC) is the standard of care for locally advanced breast cancer. For triple negative breast cancer, which is characterized by the absence of expression of estrogen receptor (ER), progesterone receptor (PR) and human epidermal growth factor receptor 2 (HER2), it is currently the only available systemic treatment option. Approximately half of all TNBC reaches a pathological complete remission (pCR) upon NAC. However, tumors without a pCR often relapse. When distant metastases develop, prognosis is poor, with a median post recurrence survival of only one year [1, 2]. As it is impossible to predict which patients will respond and which ones will not, predictive biomarkers are urgently needed. The same is true for targeted treatments, particularly for non-responding tumors.

With the introduction of next generation sequencing (NGS), tumor collections have been sequenced to categorize mutations and to find new treatment targets [3–5]. Many new mutations and several important signaling pathways have been identified. However, one of the main findings of the sequencing efforts is the marked heterogeneity within breast cancer. This heterogeneity is highlighted by the fact that recurrent mutations are infrequent. No two tumors have the same molecular make up. Consequently, subsequent studies attempting to identify broad mechanisms of chemotherapy resistance are not likely to be successful. There are many different routes to tumorigenesis, and each tumor has its own sequence of tumor-causing molecular events. The emergence of drug resistance may be driven by additional mutations and add a further level of complexity. Clinical data are not available for most studies, precluding the integration of molecular and clinical data. In addition, most existing molecular studies have been undertaken on a broad mix of breast cancer samples, with widely varying clinical stage or molecular subtype. A more homogeneous study population with well-characterized clinical data may be required to find clinical predictive factors.

To find mutations predicting chemotherapy benefit in TNBC, we performed NGS in a clinically well-characterized cohort. We sequenced an NGS panel covering 1,977 genes involved in tumorigenesis and treatment. We sequenced pretreatment biopsies from patients with a good response and patients with a poor response, to identify chemotherapy predictive markers. The purpose of this study was twofold: 1) to find chemotherapy response-predictive biomarkers and 2) to identify new treatment targets.

## Methods

### Patients and tumor biopsies

Pre-treatment biopsies and peripheral blood samples were obtained from 56 patients with untreated, primary triple negative breast cancer. All patients had received neoadjuvant treatment at the Netherlands Cancer Institute between 2004 and 2013 as part of ongoing clinical trials, or were treated off protocol according to the standard arms of one of these studies [6] (NCT00448266, NCT01057069). The ethical committee of the Netherlands Cancer Institute approved the studies and all patients gave informed consent (reference numbers of Ethical approval: PTC06.1725/N06IAA and PTC09.2716/M09TNM). All tumors were either at least 3 cm in size, or the presence of axillary lymph node metastases had been established by fine needle aspiration or pre-treatment sentinel node biopsy. Biopsies were taken using a 14-G core needle under ultrasound guidance. After collection, specimens were snap-frozen in liquid nitrogen and stored at −80 °C. Each patient had two or three biopsies taken to ensure that enough tumor material was available for both diagnostic and research purposes.

All patients started NAC with three courses of 2-weekly administrations of cyclophosphamide and doxorubicin (ddAC). The selection of the chemotherapy regimen for the fourth to the sixth courses depended on the specific clinical trial and could consist of continuation of ddAC in the case of a favorable magnetic resonance imaging (MRI) response (for criteria see previous publication [7]), randomization to intensive carboplatin-based chemotherapy or a switch to a combination of capecitabine and docetaxel in the case of an unfavorable MRI response. All patients subsequently underwent surgery, either breast conserving or mastectomy. The clinical results of these strategies have not been published because the studies are ongoing (NCT00448266, NCT01057069).

### Pathology

Triple negative status was defined by the absence of ER and PR expression and no amplification of HER2. ER (Roche Diagnostics cat. no. 5278406001 (Roche, Basel, Switserland))- and PR (Roche Diagnostics cat. no. 5278392001)-negativity was defined as immunohistochemical (IHC) staining of fewer than 10 % of tumor cell nuclei. None of the samples in this cohort had between 1 % and 10 % positive tumor cell nuclei, so all samples fulfilled the ASCO/CAP criteria for triple negativity. Negativity for HER2 (Roche Diagnostics cat. no. 5278368001) amplification was present if IHC staining was graded as 0 or 1+. In the case of 2+ or 3+ staining, chromogenic in situ hybridization was performed to determine HER2 amplification (gene copy number ≥6 per tumor cell). Chemotherapy response was assessed by microscopic examination of the surgery resection specimen. The complete absence of any invasive tumor cells in both the breast and the lymph nodes was considered as a pCR. All other responses were assigned to the no-pCR group. An experienced breast cancer pathologist (JW) reviewed all pathology slides.

### BRCA analysis

The series was well-characterized for BRCA function. Briefly, germ line DNA was isolated from peripheral blood lymphocytes of affected patients. We used mutation-scanning methods. The protein truncation test was used for exon 11 of *BRCA1* and exons 10 and 11 of *BRCA2*. The remaining exons were tested using denaturing gradient gel electrophoresis. Confirmation of aberrant samples was done by Sanger sequencing [8]. In addition, multiplex ligation-dependent probe amplification (MLPA) was performed using MLPA kit P087 (*BRCA1*) to detect large genomic deletions or duplications in the genes.

Hypermethylation of the *BRCA1* promoter was determined using methylation-specific MLPA analysis, according to the manufacturer’s protocol (MLPA assay ME005-custom, MRC-Holland, Amsterdam, The Netherlands). For normalization and analysis the Coffalyzer program was used (MRC-Holland). According to the manufacturer’s protocol, we used a cutoff of 20 % to call a sample methylated [9]. Employing this cutoff, methylated samples have very low *BRCA1* gene expression [10, 11], which was also true for this series (Additional file 1: Figure S1).

### Library preparation

DNA was isolated from frozen biopsies with a minimal tumor percentage of 50 %. Tumor cell percentage was assessed by an experienced breast pathologist (JW). Isolation was performed with (Qiagen, Venlo, the Netherlands) DNA mini kit. Matched normal DNA was obtained from peripheral blood and extracted by DNAzol and purified with Qiagen DNeasy kit. Samples were interrogated by a designed “Cancer mini-genome” consisting of 1,977 cancer genes [12]. This set comprises genes involved in DNA repair, cell cycle, apoptosis, epigenetic modification (methylation, acetylation), angiogenesis (vascular endothelial growth factor (VEGF) pathway), and genes from the epidermal growth factor receptor (EGFR), mammalian target of rapamycin (mTOR), insulin, TP53, transforming growth factor (TGF)-beta, Notch, Wnt and hedgehog pathways, known tumor suppressor and proto-oncogenes, and all additional kinases not present in any of the above mentioned processes. Genes are listed in Additional file 2: Table S1. Barcoded fragment libraries were generated from 300–600 ng of isolated DNA from tumor and reference samples as previously described [13]. Pools of libraries were enriched for 1,977 cancer-related genes using (SureSelect technology, Agilent, Santa Clara, California, US). Enriched libraries were sequenced to achieve an average coverage of at least 150× on a (SOLiD 5500xl, Applied Biosystems, Waltham, Massachusetts, US) instrument according to the manufacturers’ protocol. Sequencing statistics are available in Additional file 3: Table S2.

### Sequence data analysis

Sequence reads were mapped on the human reference genome version 19 (GRCh37), using BWA (−c –l 25 –k 2 –n 10) [14] and variant calling was done using a custom pipeline identifying variants with at least 10× coverage, a 20 % allele frequency, and multiple (> = 2) occurrences in the seed (the first 25-bp most accurately mapped part of the read) and support from independent reads (> = 3). Validation of our custom variant-calling pipeline is described in Nijman et al. [15]. Putative somatic variants were identified by subsequently genotyping all variant positions in the raw datasets of both the tumor and reference sample using samtools mpileup, to ensure the absence of variant alleles in the reference sample; only positions showing less than 5 % variant alleles in the reference sample were considered somatic. Next, somatic variants with a minor allele frequency >0.01 in non-*Catalogue of Somatic Mutations in Cancer* (COSMIC) populations in dbSNP were filtered out, as were variants seen > = 1× homozygously or > = 3× heterozygously in reference samples in our in-house database and variants with an allele frequency <25 % if the coverage was <50×. Finally, manual curation was performed to remove obvious noise in poorly mapped regions. For the analysis of association with clinical data and the pathway analysis, we included only putative driver mutations defined as either 1) mutations with a major effect on protein expression, defined as a) truncating mutations, indels, splice acceptors/donors, initiator codon variants or b) non-synonymous mutations predicted to be damaging according to PolyPhen [16] and/or SIFT [17], or 2) known COSMIC mutations.

Copy number analysis was done by comparing depth of coverage in reference and tumor samples using the robust *z* score according to the method of Iglewicz and Hoaglin [18]. Briefly, the median and median absolute deviation of coverage per exon of all reference samples, normalized on the total number of reads per sample, was calculated to determine the coverage distribution. The *z* -scores (i.e., the difference between the coverage of the tumor sample and the median coverage of the reference pool of normal samples, multiplied by a correction factor of 0.6745, and divided by the median absolute deviation of the reference pool) were calculated for all exons of each tumor sample. The *z* score of the total gene was calculated by taking the average of the *z* scores per exon. As we noticed an effect of GC-content on the exon-coverage profiles in some, but not all samples, we used unsupervised hierarchical clustering of the normalized coverage per exon of all samples. Two clear clusters were formed, but 12 samples (including both tumor and reference samples) could not be placed in either cluster. Exon coverage of the tumor samples was then compared to the reference samples within the same cluster. Genes with *z* scores > = 10 were considered high-level amplifications; a *z* score > = 3 indicates copy number gain and < = −3 copy number loss.

### Sanger sequencing for validation of PIK3CA mutations

Samples were analyzed by Sanger Sequencing using a BigDye Terminator Cycle Sequencing Kit and ABI 3730 capillary sequencer (Life Technologies/Applied Biosystems, Waltham, Massachusetts, US).

### Statistics: association of sequencing data with clinical information

Statistical analyses were performed with R/Bioconductor [19]. We focused on three clinical variables: 1) pCR, 2) relapse and 3) BRCA proficiency. We encoded each of the clinical variables into a binary variable and then studied associations at the gene and at the pathway levels: association between mutations in an individual gene and the given clinical variable was tested using Fisher’s exact test of the presence/absence of a mutation in a sample and the presence/absence of the clinical variable. The pathway analysis was based on a list of 29 pathways from the Kyoto Encyclopedia of Genes and Genomes (KEGG), including at least three genes targeted in the sequencing experiments. We considered a pathway to be mutated if at least one of its genes was found mutated. We then performed the Fisher’s exact test of the presence/absence of a mutation in a given pathway and the presence/absence of the clinical variable in the various samples. All tests were corrected for multiple testing using the Benjamini-Hochberg method. In all analyses, associations were considered significant when the adjusted *p* value was <0.05.

## Results

### Description of the dataset

We performed deep sequencing on a set of fifty-six triple negative pre-treatment biopsies, scheduled to receive NAC. The pathological complete remission rate was 45 % (Table 1). With a median follow up of 2.5 years, nine patients had a relapse (16 %). Twelve patients had a *BRCA1* or *BRCA2* germline mutation, and seventeen biopsies showed *BRCA1* promoter methylation. In total, BRCA function (based on either a *BRCA1/2* mutation or *BRCA1* promoter methylation) was impaired in at least 29 tumors (52 %). This number could be higher as *BRCA1* germline screening was not performed for five cases, as these cases did not qualify for routine diagnostic *BRCA1* and *BRCA2* germline testing (based on age, family history). Between 1 and 36 somatic mutations per sample were detected (10 mutations on average; Additional file 4: Table S3).

**Table 1:** Patient and tumor characteristics

Characteristic	Values (n = 56 patients)
Age	
Median, range	40 (23–74)
Tumor stage	
1	5 (9)
2	40 (71)
3	7 (13)
4	4 (7)
Nodal stage	
Lymph nodes negative	19 (34)
Lymph nodes positive	37 (66)
Grade	
II	18 (32)
III	34 (61)
Not determined	4 (7)
Chemotherapy	
6 × dose dense doxorubicin/cyclophosphamide	43 (77)
3 × dose dense doxorubicin/cyclophosphamide, 3 × docetaxel/capecitabine	5 (9)
4 × dose dense doxorubicin/cyclophosphamide, 2 × cyclophosphamide/thiotepa/carboplatin	8 (14)
BRCA germline mutation status (Sanger sequencing)	
*BRCA1* mutation	10 (18)
*BRCA2* mutation	2 (4)
Wildtype	39 (70)
Not determined	5 (9)
*BRCA1* promoter methylation	
No *BRCA1* methylation	39 (70)
*BRCA1* methylation	17 (30)
Response breast and lymph nodes	
No pathological complete remission	31 (55)
Pathological complete remission	25 (45)
Relapse	
No relapse	47 (84)
Relapse	9 (16)

Values are n (%) unless stated otherwise

### Few recurrent mutations in TNBC

**Fig. 1:**
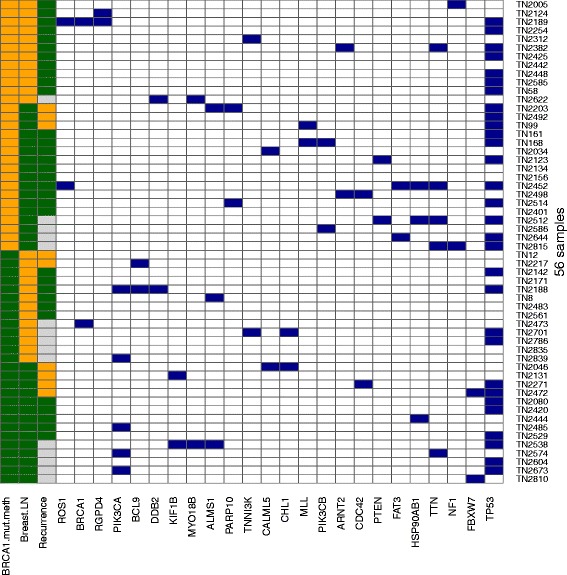
Heatmap of mutations occurring in two or more samples. *Blue box* mutation; *left* the three clinical variables; *yellow* subsequently a *BRCA1* mutation or methylation, a pathological complete remission (pCR), and a recurrence. *Gray* samples with follow up time that was not long enough to be included in the relapse analysis

The somatic mutation rates were similar for responders and non-responders (average mutation rate of nine versus eight mutations per tumor, *p* = 0.32). The mutation spectrum was broad with few recurrent mutations in the dataset. Figure 1 shows the mutations occurring in three or more samples. Mutations in *TP53*, *TTN* and *PIK3CA* were the most frequently observed alterations in this study (55 %, 14 %, and 9 %, respectively). Potentially dubious genes were extracted from Lawrence et al. [20] and indicated as such in the mutation data (Additional file 4: Table S3).

### Association of mutations with clinical variables

There were no putative driver mutations significantly associated with either chemotherapy response (pCR rate) or with relapse (Additional file 5: Table S4). An explanation could be the heterogeneous nature of TNBCs and lack of recurrent mutations in TNBCs.

Next, we assessed whether any putative driver mutations were associated with BRCA deficiency, either resulting from a germline mutation or from promoter methylation. *PIK3CA* mutations were exclusively observed in patients with functional BRCA genes (5/27 (19 %)) versus 0/29 in the patients with a *BRCA* mutation or *BRCA1* promoter methylation (*p* = 0.02, adjusted *p* value (*p*-adj) = 0.11) (Table 2). Tumors with BRCA impairment may develop via different routes than tumors with functioning BRCA genes, and the PI3K pathway may play a role in the latter. This finding has also been recently observed in an independent dataset [21]. All *PIK3CA* mutations were confirmed with Sanger sequencing.

**Table 2:** Association of mutations with BRCA proficiency

Gene	BRCA mutated/methylated (number)	BRCA proficient (number)	BRCA mutated/methylated, and mutated (number)^a^	BRCA proficient and mutated (number)^a^	*P* value	Adjusted *p* value
*PIK3CA*	29	27	0	5	0.02	0.11
*TP53*	29	27	18	12	0.28	0.59
*TTN* ^b^	29	27	4	1	0.35	0.59
*ALMS1*	29	27	1	2	0.60	0.76
*HSP90AB1*	29	27	2	1	1.00	1.00

^a^All samples with a BRCA mutation/methylation and a mutation in the respective gene, and applies likewise to the BRCA proficient and mutated column, Table 3, and Table 4. ^b^The *TTN* gene is often mutated due to its greater length and may not be cancer-related (see also Additional file 4: Table S3)

### Pathway analysis

We next investigated whether mutations in different genes would cluster in some known pathways. Using KEGG, we derived a pathway mutation status based on its gene members (see “Methods”) and looked for association with the clinical variables (Table 3; Additional file 6: Table S5 and Additional file 7: Table S6). Calcium signaling was inversely associated with a pCR (*p* = 0.03), as was Notch signaling (*p* = 0.03). BRCA mutated or methylated tumors were associated with mutations in the Wnt signaling pathway (*p* = 0.06). However, after correcting for multiple testing, none of the associations remained statistically significant. No pathways were associated with relapse.

**Table 3:** Association of KEGG pathways with clinical variables

A. Pathological complete remission (pCR)								
Pathway	Genes in pathway	Genes mutated	pCR (number)	No pCR (number)	pCR and mutated (number)	No pCR and mutated (number)	*P* value	Adjusted *p* value
Calcium signaling pathway	177	6	25	31	0	6	0.03	0.41
Notch signaling pathway	47	4	25	31	4	0	0.03	0.41
TGF-beta signaling pathway	85	6	25	31	0	5	0.06	0.41
Phosphatidylinositol signaling system	78	11	25	31	4	12	0.08	0.41
Focal adhesion	200	25	25	31	7	16	0.10	0.41
B. BRCA proficiency								
Pathway	Genes in pathway	Genes mutated	BRCA mutated/methylated (number)	BRCA proficient (number)	BRCA mutated/methylated, and mutated (number)	BRCA proficient and mutated (number)	*P* value	Adjusted *p* value
Wnt signaling pathway	151	12	29	27	21	12	0.06	0.78
Jak-STAT signaling pathway	155	3	29	27	2	6	0.14	0.78
Regulation of actin cytoskeleton	214	16	29	27	6	11	0.15	0.78
MAPK signaling pathway	268	28	29	27	21	14	0.17	0.78
mTOR signaling pathway	52	5	29	27	3	7	0.17	0.78

*TGF* transforming growth factor, *MAPK* mitogen activated protein kinase, *mTOR* mammalian target of rapamycin

### Copy number analysis

Fifty out of fifty-six tumors had NGS data suitable for copy number analysis. We identified high-level, focal amplifications of several growth factor receptors such as *EGFR* and *FGFR2*, and amplification of *AIM1* in three samples, *MET* amplification in three samples and *CCNE1* and *PIK3CA* in single cases (Additional file 8: Figure S2). Multiple focal, high-level amplifications were visible in several samples, affecting up to five different chromosomes per patient, while other samples showed no such events. We also detected recurrent gains of cyclins (*CCND1*, *CCND2*), *MYC* and *FGFR1*. Chromosome 1q and 8q (including MYC) showed gains in several samples, both events were frequently detected in the same sample.

Patients with a relapse or without a pCR in the breast had a higher number of genes with copy number alterations (CNA) than patients without a relapse or with a pCR in the breast (*p* = 0.0091 and *p* = 0.023, respectively). Specific amplifications and deletions were associated with response, relapse and BRCA proficiency. Most interesting is that *TTK* and *TP53BP2* amplifications were associated with a poor response (*p* = 0.02 and *p* = 0.06, respectively). However, after correction for multiple testing, the *p* values were no longer significant. Table 4 gives an overview of the CNAs related to the clinical phenotypes. *TTK* is involved in mitotic checkpoint, and over-expression was recently reported to be associated with TNBC. *TTK* might be an attractive therapeutic target [22]. *TTK* and *TP53BP2* amplifications were also observed in Nimblegen copy number data of the same samples (Additional file 9: Figure S3).

**Table 4:** Association of copy number variations with clinical variables

A. Pathological complete remission (pCR)						
Gene	pCR (number)	No pCR (number)	pCR and CNV (number)	No pCR and CNV (number)	*P* value	Adjusted *p* value
*TTK*	23	28	0	8	0.02	0.95
*TP53BP2*	23	28	4	15	0.06	0.95
*SNAI2*	23	28	1	8	0.09	1.00
*STK3*	23	28	1	8	0.09	1.00
*ABL2*	23	28	7	3	0.11	1.00
B. Relapse						
Gene	Relapse (number)	No relapse (number)	Relapse and CNV (number)	No relapse and CNV (number)	*P* value	Adjusted *p* value
*SNAI2*	8	32	4	1	0.03	0.14
*MAPK15*	8	32	5	3	0.06	0.14
*TTN*	8	32	0	9	0.07	0.65
*PARP10*	8	32	6	4	0.08	0.14
*STYK1*	8	32	0	7	0.11	0.77
C. BRCA proficiency						
Gene	BRCA mut/meth (number)	BRCA proficient (number)	BRCA mut/meth and CNV (number)	BRCA proficient and CNV (number)	*P* value	Adjusted *p* value
*IFT81*	27	24	1	8	0.001	0.36
*SNAI2*	27	24	1	8	0.01	0.36
*TNFAIP3*	27	24	9	0	0.01	0.88
* PLCXD3*	27	24	5	11	0.02	1.00
*CCNB2*	27	24	10	1	0.06	1.00

*mut/meth* mutated/methylated, *CNV* copy number variation

## Discussion

In this study we deep-sequenced a cohort of 56 pre-treatment breast cancer biopsies to find chemotherapy predictive biomarkers and new treatment targets. We found that TNBC was very heterogeneous with few recurrent mutations. As a result, we were not able to find biomarkers associated with chemotherapy response and relapse. We found that tumors without BRCA impairment (through a mutation or through *BRCA1*-promoter methylation) frequently had *PIK3CA* mutations.

The heterogeneous nature of TNBC has already been described by others [3, 5, 23] and makes it difficult to find predictive biomarkers. There may well be hundreds of different mutations associated with resistance to standard chemotherapy. This fact would explain why overall comparison of groups of sensitive versus resistant tumors does not lead to candidate markers. The same problem exists for gene expression studies: due to several factors, among which the highly variable makeup of tumors may be central, it has proven to be extremely difficult to find predictive profiles [24]. Different genes may explain resistance in subgroups of tumors as we have shown for HER2-negative tumors in our gene expression studies [25, 26]. A possibility to analyze NGS mutation data in a different manner is to investigate logical combinations of the mutation status of several genes and how such a combination relates to a clinical outcome variable of interest. For instance, mutation in gene A and not in gene B could be predictive of response. However, the number of possible combinations is so large that such an analysis is not practical with datasets of the size presented here. In this study we have used external knowledge (KEGG pathways) to focus on the combination of some specific subsets of genes. It would be interesting to investigate combinations of genes beyond these annotated pathways.

Previous research by our group and by others has shown that a proportion of TNBC shares similarities with *BRCA1*-mutated breast cancers [10, 27–29]. This phenotype is often called “BRCAness”. As BRCA1 and BRCA2 are involved in the repair of DNA double-strand breaks (DSBs), a process called homologous recombination, dysfunctional BRCA could render a tumor hypersensitive to drugs that (ultimately) induce DNA DSBs during the DNA replication phase, such as the PARP inhibitors or alkylating chemotherapy. In this study we observed that 22 % of the tumors had a BRCA mutation and 30 % had *BRCA1* promoter methylation, indicating that BRCA inactivation is observed in over half of the tumors. *PIK3CA* mutations were only observed in the tumors proficient for BRCA. Our group has been extensively studying tumors with *BRCA1* promoter methylation or a BRCA1-like genomic profile. We found that these BRCA1-like tumors resemble *BRCA1* mutation carriers in multiple respects, i.e., age, grade and good chemotherapy response [29]. Most importantly those BRCA1-like tumors have an excellent response to DSB-inducing chemotherapy [30]. The association between non-BRCA1-like tumors and *PIK3CA* mutations was also seen in an independent study [21]. Other studies found that *PIK3CA* mutations are more frequent in HER2+ and luminal breast cancer; it might be that we defined a subgroup of TNBCs where *PIK3CA* mutations are also frequent, i.e., the non-BRCA subgroups. Another consequence of this finding could be that some non-BRCA-related tumors could be susceptible to PIK3CA inhibitors.

As far as we know this is the first deep sequencing study comparing well-responding and poorly-responding breast tumors in the neoadjuvant setting to identify biomarkers. Balko et al. [31] performed NGS on TNBC after neoadjuvant treatment. This study focused on a cohort of cancers with macroscopic residual disease after NAC, which is associated with poor survival. When we compare our results with the Balko study we see the following. The Balko study detected a higher frequency of several targetable alterations when comparing data with The Cancer Genome Atlas (TCGA) Breast [3]. These alterations included *MCL1*, *PTEN*, and *JAK2*. As expected, the percentage of these mutations in our study was more comparable to the Balko data than to TCGA (Additional file 10: Table S7). Whereas the Balko dataset contained only samples that had been taken after neoadjuvant chemotherapy, the TCGA cohort contains all clinical breast cancer stages. However, the Balko series only consists of tumors with a poor response to chemotherapy response, whereas our series consists of both responding and non-responding tumors. We also detected a high percentage of specific copy number alterations, like *DDR2*, *PYGO2* and *SCYL3*. As the Balko study sequenced only 182 oncogenes, those genes were not in their sequencing panel.

A limitation of this study is the small number of samples with relapse. This fact limits the power and reduces the likelihood of finding associations with relapse. However, if there were strong associations with relapse they would have been picked up.

Although our dataset is small with 56 tumors, and precludes us from making any firm conclusions about markers associated with neoadjuvant chemotherapy response in TNBC, we can draw several potentially important conclusions. First, the mutation spectrum remains diverse even in a carefully selected and untreated group of patients with TNBC from the same clinical stage (locally advanced breast cancer, i.e., larger than 3 cm and/or positive lymph nodes). In addition, all samples were from the same institute and the laboratory procedures were thoroughly monitored: tumor percentage was 50 % or more, a matching normal DNA sample was available for every tumor, and pathology assessment of the whole series had been performed by a single specialized breast pathologist (JW). Our results strongly suggest that every tumor has its unique molecular makeup. Second, we found that *PIK3CA* mutations were specifically associated with *BRCA1* proficient tumors, a finding that was also observed in another study [21]. This finding may have clinical consequences. This study shows that it is increasingly important to have large series of homogenous sample series, with clinically well-annotated data.

## Conclusions

Two important conclusions can be drawn from these data. First, TNBC is heterogeneous. Even in a homogenous, clinically well-annotated sample series, few recurrent mutations can be identified. Second, *PIK3CA* mutations were associated with BRCA proficient tumors. After validation in larger series, triple negative tumors with *PIK3CA* mutations can be candidates for agents targeting these oncogenic pathways. To identify recurrent mutations associated with chemotherapy or relapse, larger sample series are urgently needed.

## Additional files

Additional file 1: Figure S1.
*BRCA1* gene expression, measured by RT-PCR, for *BRCA1*-methylated, mutated and non-mutated/non-methylated triple negative breast cancer (*TNBC*) samples. This figure shows the association between BRCA proficiency/deficiency and *BRCA1* gene expression, as measured by RT-PCR. Samples with either *BRCA1* promoter methylation or a *BRCA1* mutation all had low gene expression values. (DOCX 134 kb) Additional file 2: Table S1. 1977 Cancer Gene Panel. This file lists all 1,977 genes in the Cancer Gene Panel, used in this study. (XLS 111 kb) Additional file 3: Table S2. Overview of sequencing statistics. (XLSX 48 kb) Additional file 4: Table S3. List of all mutations. This file shows all mutations passing thresholds as explained in “Methods”. The column “confirmed” shows mutations that have been confirmed by either Sanger Sequencing or deep whole exome sequencing in another research project (unpublished data). The column “included in analysis” shows the mutations that passed our threshold for putative driver mutations, based on effect, SIFT, polyphen scores and COSMIC (see “Methods”). The column “dubious” shows the mutations that might be dubious based on gene length, expression or replication time [20]. (XLSX 64 kb) Additional file 5: Table S4. Association between mutations and chemotherapy response or relapse. This table shows the top five genes associated with either response or relapse. No significant associations were found. (DOCX 18 kb) Additional file 6: Table S5. Association between relapse and pathway mutations. This table shows the top five pathways associated with relapse. No significant associations were found. (DOCX 14 kb) Additional file 7: Table S6. Specific mutations within affected pathways. This file shows all pathways with KEGG IDs, and mutations affecting the different pathways. (XLSX 10 kb) Additional file 8: Figure S2. Heatmap of all copy number aberrations in the dataset. Samples are represented by *rows*, genomic regions by the *columns* sorted on genomic order. *Red* identifies a high level gain or amplification; *blue* a loss. (PNG 130 kb) Additional file 9: Figure S3. Validation of *TTK*, *TP53BP2* and genome-wide copy number profiles with Nimblegen 135 K aCGH arrays. aCGH and exome data, for the samples for which aCGH data were available (three cases for *TTK*, five cases for *TP53BP2*). *Arrow* indicates respectively the *TTK* and *TP53BP2* locus. From page 3 on we show all 14 samples for which we have both exome and aCGH data, irrespective of *TTK* and *TP53BP2* status. In these rainbow plots, the samples containing a *TTK* or *TP53BP2* gain are indicated with an *arrow* at the respective genomic location. (PDF 1639 kb) Additional file 10: Table S7. Comparison of top alterations listed by Balko et al. (reference [30]) (A) and our study (B) for TCGA [3], Balko et al. [31] and our study. Part A shows the top alterations as listed by Balko (reference [30]) in our study and TCGA (reference [3]), part B shows the top alterations in our study. (DOCX 17 kb)

## Abbreviations

bp: base pairs
CNA: copy number alteration
COSMIC: Catalogue of Somatic Mutations in Cancer
ddAC: dose dense cyclophosphamide, doxorubicin
DSB: double strand break
EGFR: epidermal growth factor receptor
ER: estrogen receptor
HER2: human epidermal growth factor receptor 2
KEGG: Kyoto Encyclopedia of Genes and Genomes
MLPA: multiplex ligation-dependent probe amplification
mTOR: mammalian target of rapamycin
NAC: neoadjuvant chemotherapy
NGS: next generation sequencing
pCR: pathological complete remission
PR: progesterone
TGF: transforming growth factor
TNBC: triple negative breast cancer
VEGF: vascular endothelial growth factor
