# Supplementary material for: Next generation sequencing of triple negative breast cancer to find predictors for chemotherapy response
Source: Breast Cancer Res. 2015 Oct 3;17:134. doi: 10.1186/s13058-015-0642-8 (PMC4592753; doi:10.1186/s13058-015-0642-8)
Supplement: Additional file 1: Figure S1. — BRCA1 gene expression, measured by RT-PCR, for BRCA1-methylated, mutated and non-mutated/non-methylated triple negative breast cancer (TNBC) samples. This figure shows the association between BRCA proficiency/deficiency and BRCA1 gene expression, as measured by RT-PCR. Samples with either BRCA1 promoter methylation or a BRCA1 mutation all had low gene expression values. (DOCX 134 kb) [file 13058_2015_642_MOESM1_ESM.docx]

Figure S1. BRCA1 gene expression, measured by RT-PCR, for BRCA1-methylated, mutated and non mutated/methylated TNBC samples.
