# Supplementary material for: Next generation sequencing of triple negative breast cancer to find predictors for chemotherapy response
Source: Breast Cancer Res. 2015 Oct 3;17:134. doi: 10.1186/s13058-015-0642-8 (PMC4592753; doi:10.1186/s13058-015-0642-8)
Supplement: Additional file 5: Table S4. — Association between mutations and chemotherapy response or relapse. This table shows the top five genes associated with either response or relapse. No significant associations were found. (DOCX 18 kb) [file 13058_2015_642_MOESM5_ESM.docx]

**Table S4. Association of identified mutations with chemotherapy response (pathological remission) and relapse (for each variable the 5 genes with the lowest p-value are shown)**

| A. Pathological Complete Remission (pCR) | | |  |  |  |  |
| --- | --- | --- | --- | --- | --- | --- |
| gene | pCR (n) | no pCR (n) | pCR & mutated (n)* | no pCR & mutated (n) | pval | pval.adj |
| HSP90AB1 | 25 | 31 | 0 | 3 | 0.25 | 0.61 |
| TP53 | 25 | 31 | 11 | 19 | 0.28 | 0.61 |
| TTN | 25 | 31 | 1 | 4 | 0.37 | 0.61 |
| ALMS1 | 25 | 31 | 1 | 2 | 1.00 | 1.00 |
| PIK3CA | 25 | 31 | 2 | 3 | 1.00 | 1.00 |
| B. Relapse |  |  |  |  |  |  |
| gene | Relapse (n) | No Relapse (n) | Relapse & mutated (n) | No Relapse & mutated (n) | pval | pval.adj |
| ALMS1 | 9 | 32 | 1 | 1 | 0.40 | 1.00 |
| HSP90AB1 | 9 | 32 | 0 | 2 | 1.00 | 1.00 |
| PIK3CA | 9 | 32 | 0 | 2 | 1.00 | 1.00 |
| TP53 | 9 | 32 | 5 | 17 | 1.00 | 1.00 |
| TTN | 9 | 32 | 0 | 2 | 1.00 | 1.00 |

*pCR & mutated: This column shows all samples with a pCR and a mutation in the respective gene. This applies likewise for the other columns (‘no pCR & mutated’, ‘Relapse & mutated’, etc.) and for the other tables.
