# Supplementary material for: Next generation sequencing of triple negative breast cancer to find predictors for chemotherapy response
Source: Breast Cancer Res. 2015 Oct 3;17:134. doi: 10.1186/s13058-015-0642-8 (PMC4592753; doi:10.1186/s13058-015-0642-8)
Supplement: Additional file 6: Table S5. — Association between relapse and pathway mutations. This table shows the top five pathways associated with relapse. No significant associations were found. (DOCX 14 kb) [file 13058_2015_642_MOESM6_ESM.docx]

**Table S5. Association of KEGG pathways with relapse**

| Pathway | Genes in pathway | Genes mutated | Relapse (n) | No Relapse (n) | Relapse & mutated (n) | No Relapse & mutated (n) | pval | pval.adj |
| --- | --- | --- | --- | --- | --- | --- | --- | --- |
| Ubiquitin mediated proteolysis | 139 | 5 | 9 | 32 | 2 | 2 | 0.20 | 1.00 |
| Inositol phosphate metabolism | 57 | 8 | 9 | 32 | 0 | 7 | 0.31 | 1.00 |
| Adherens junction | 73 | 3 | 9 | 32 | 1 | 1 | 0.40 | 1.00 |
| Cytokine-cytokine receptor interaction | 265 | 5 | 9 | 32 | 1 | 2 | 0.53 | 1.00 |
| ECM-receptor interaction | 85 | 6 | 9 | 32 | 0 | 5 | 0.57 | 1.00 |
