# Supplementary figures and images for: Next generation sequencing of triple negative breast cancer to find predictors for chemotherapy response
Source: Breast Cancer Res. 2015 Oct 3;17:134. doi: 10.1186/s13058-015-0642-8 (PMC4592753; doi:10.1186/s13058-015-0642-8)

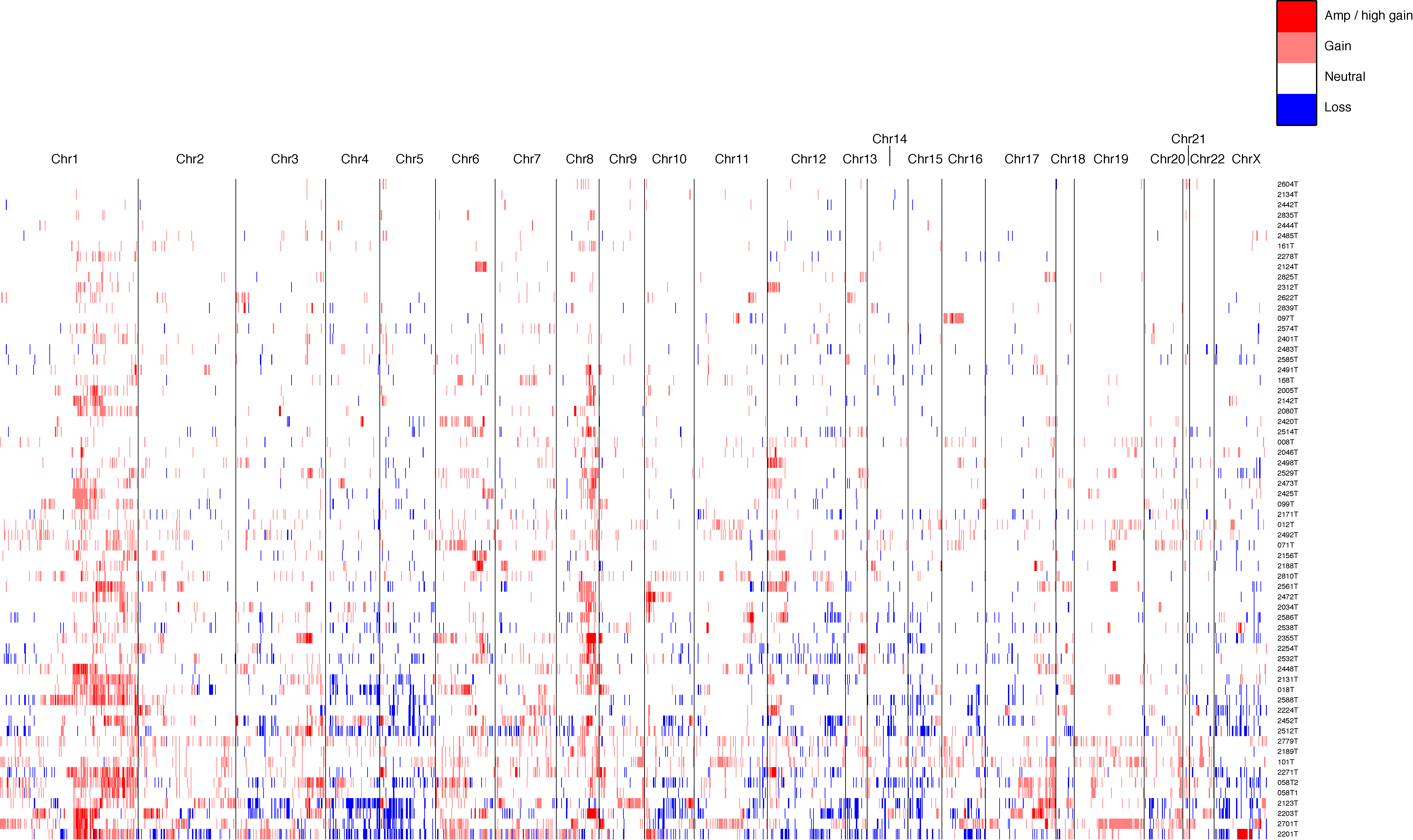

Supplement: Additional file 8: Figure S2. — Heatmap of all copy number aberrations in the dataset. Samples are represented by rows, genomic regions by the columns sorted on genomic order. Red identifies a high level gain or amplification; blue a loss. (PNG 130 kb) [file 13058_2015_642_MOESM8_ESM.png]
