# Supplementary material for: Next generation sequencing of triple negative breast cancer to find predictors for chemotherapy response
Source: Breast Cancer Res. 2015 Oct 3;17:134. doi: 10.1186/s13058-015-0642-8 (PMC4592753; doi:10.1186/s13058-015-0642-8)
Supplement: Additional file 9: Figure S3. — Validation of TTK, TP53BP2 and genome-wide copy number profiles with Nimblegen 135 K aCGH arrays. aCGH and exome data, for the samples for which aCGH data were available (three cases for TTK, five cases for TP53BP2). Arrow indicates respectively the TTK and TP53BP2 locus. From page 3 on we show all 14 samples for which we have both exome and aCGH data, irrespective of TTK and TP53BP2 status. In these rainbow plots, the samples containing a TTK or TP53BP2 gain are indicated with an arrow at the respective genomic location. (PDF 1639 kb) [file 13058_2015_642_MOESM9_ESM.pdf]

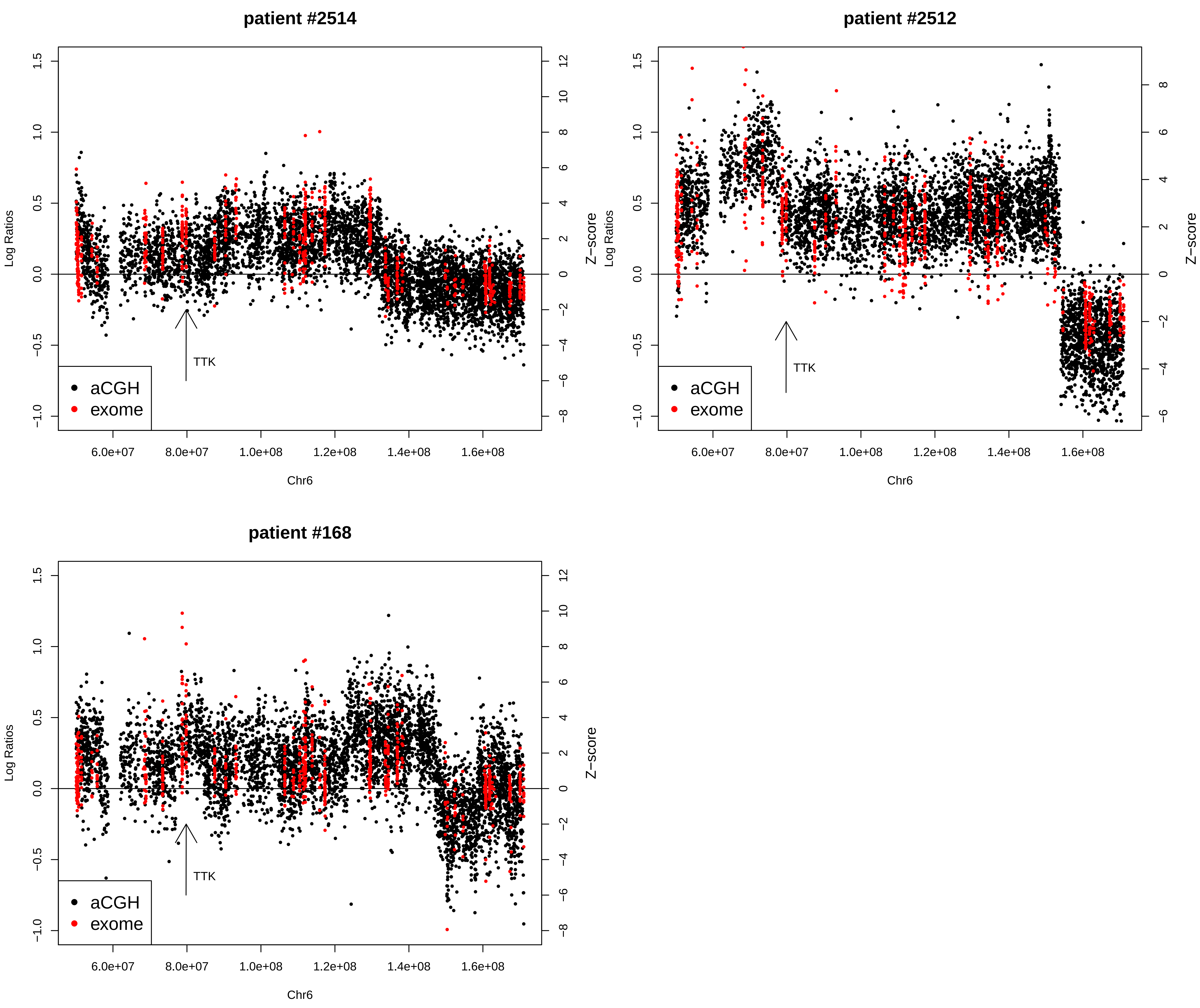
patient #2514
patient #2512
patient #168
Log Ratios
Z−score
Chr6
TTK
aCGH
exome
6.0e+07
8.0e+07
1.0e+08
1.2e+08
1.4e+08
1.6e+08

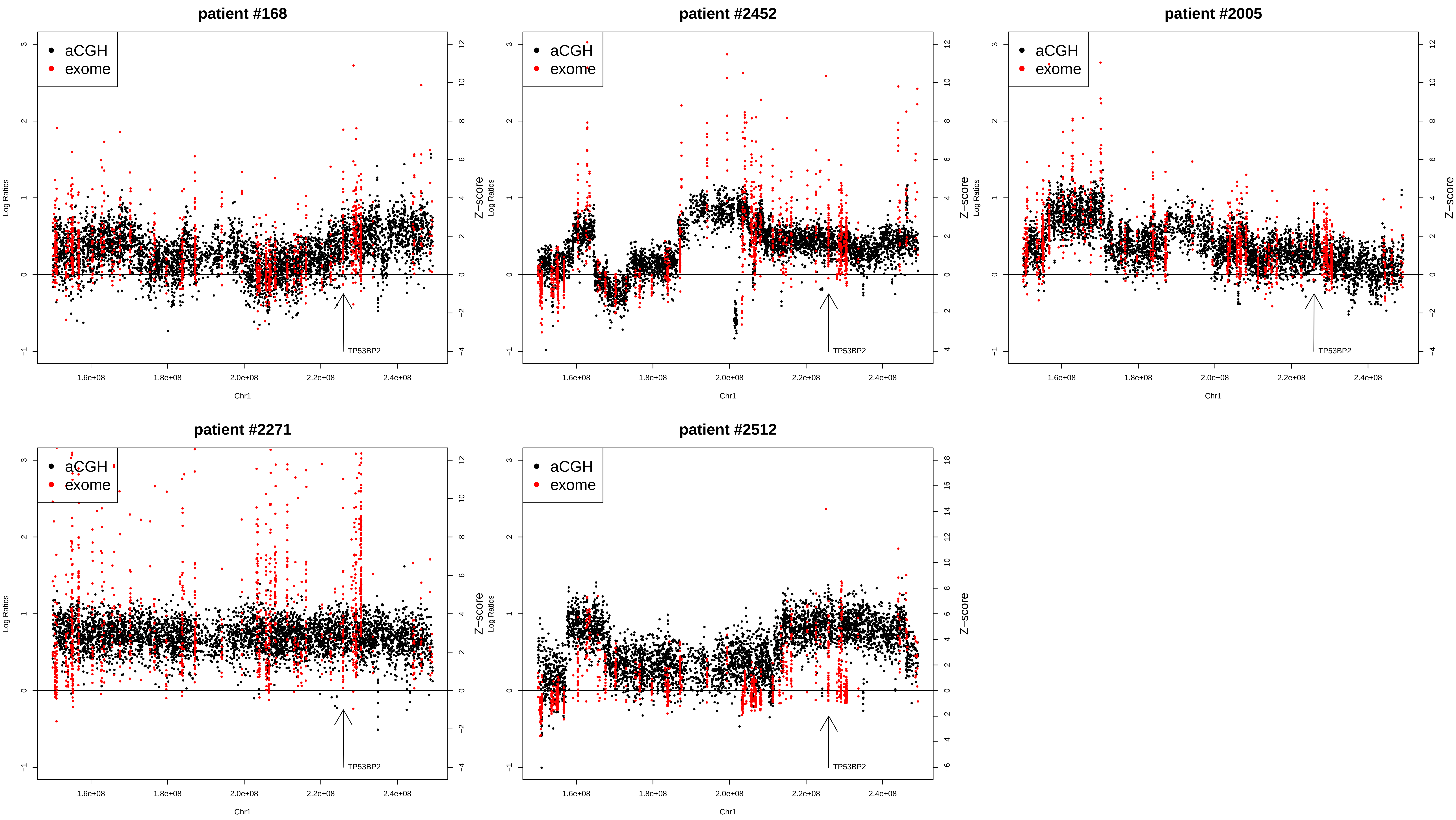
patient #168
patient #2452
patient #2005
patient #2271
patient #2512
aCGH
exome
Log Ratios
Z−score
Chr1
1.6e+08
1.8e+08
2.0e+08
2.2e+08
2.4e+08
TP53BP2

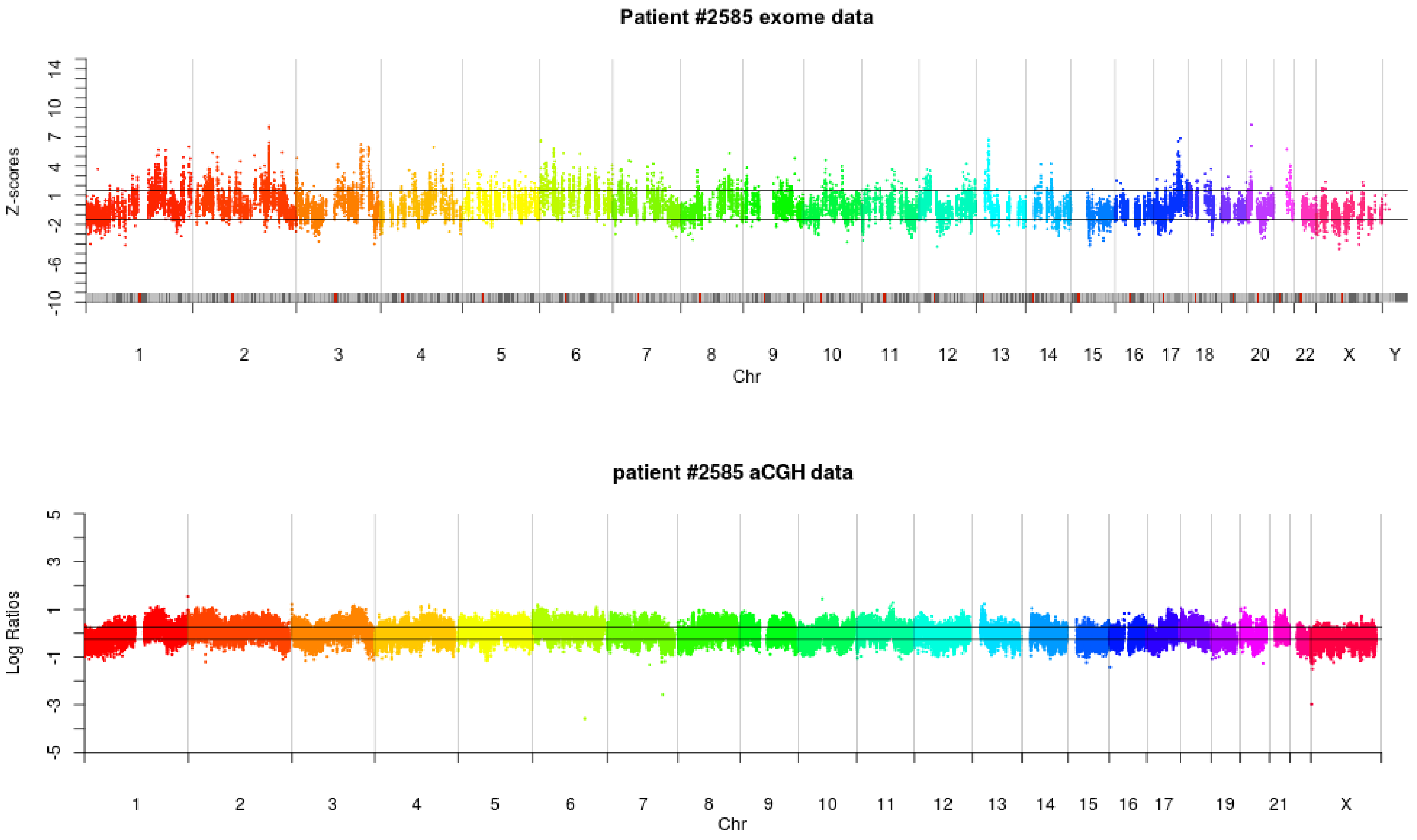

Patient #2585 exome data
Z-scores
14
10
7
4
1
-2
-6
-10
1
2
3
4
5
6
7
8
9
10
11
12
13
14
15
16
17
18
20
22
X
Y
Chr
patient #2585 aCGH data
Log Ratios
5
3
1
-1
-3
-5
1
2
3
4
5
6
7
8
9
10
11
12
13
14
15
16
17
19
21
X
Chr

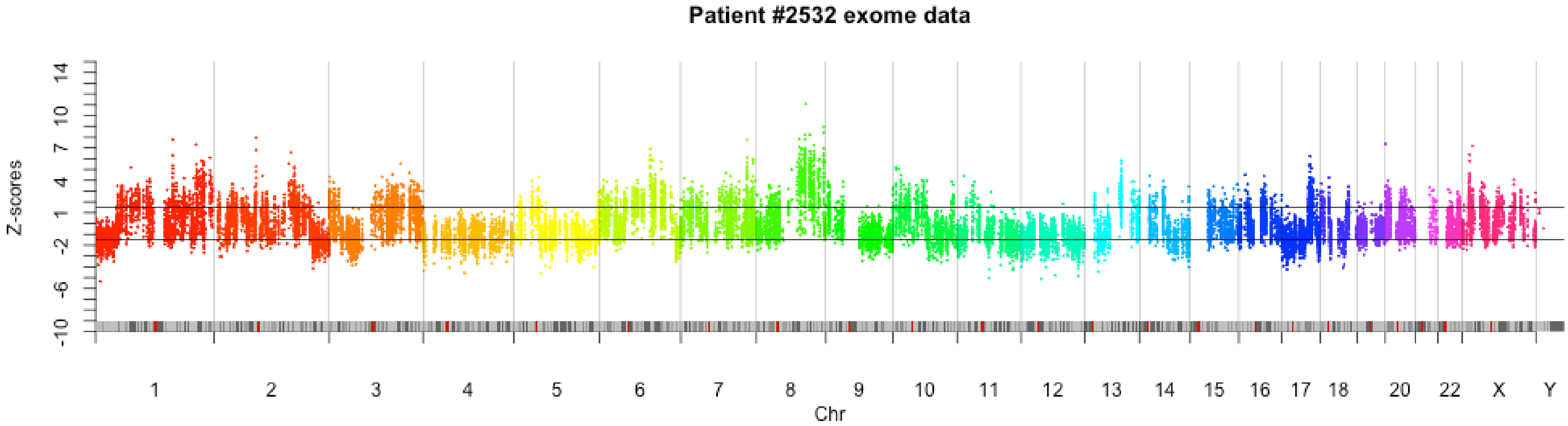
Patient #2532 exome data
Z-scores
14
10
7
4
1
-2
-6
-10
1
2
3
4
5
6
7
8
9
10
11
12
13
14
15
16
17
18
20
22
X
Y
Chr

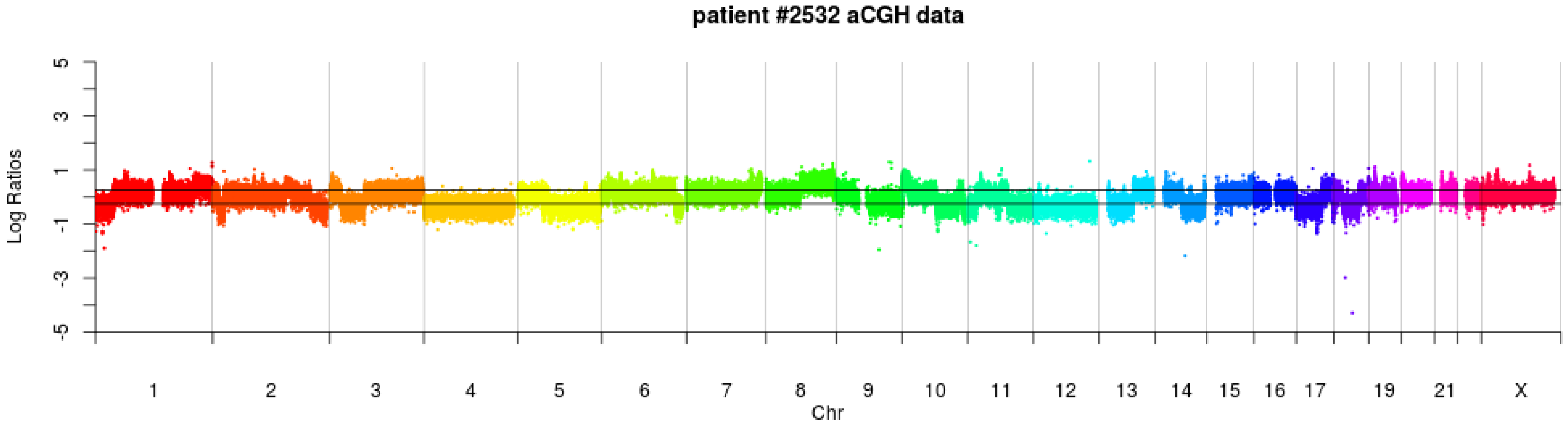
patient #2532 aCGH data
Log Ratios
5
3
1
-1
-3
-5
1
2
3
4
5
6
7
8
9
10
11
12
13
14
15
16
17
19
21
X
Chr

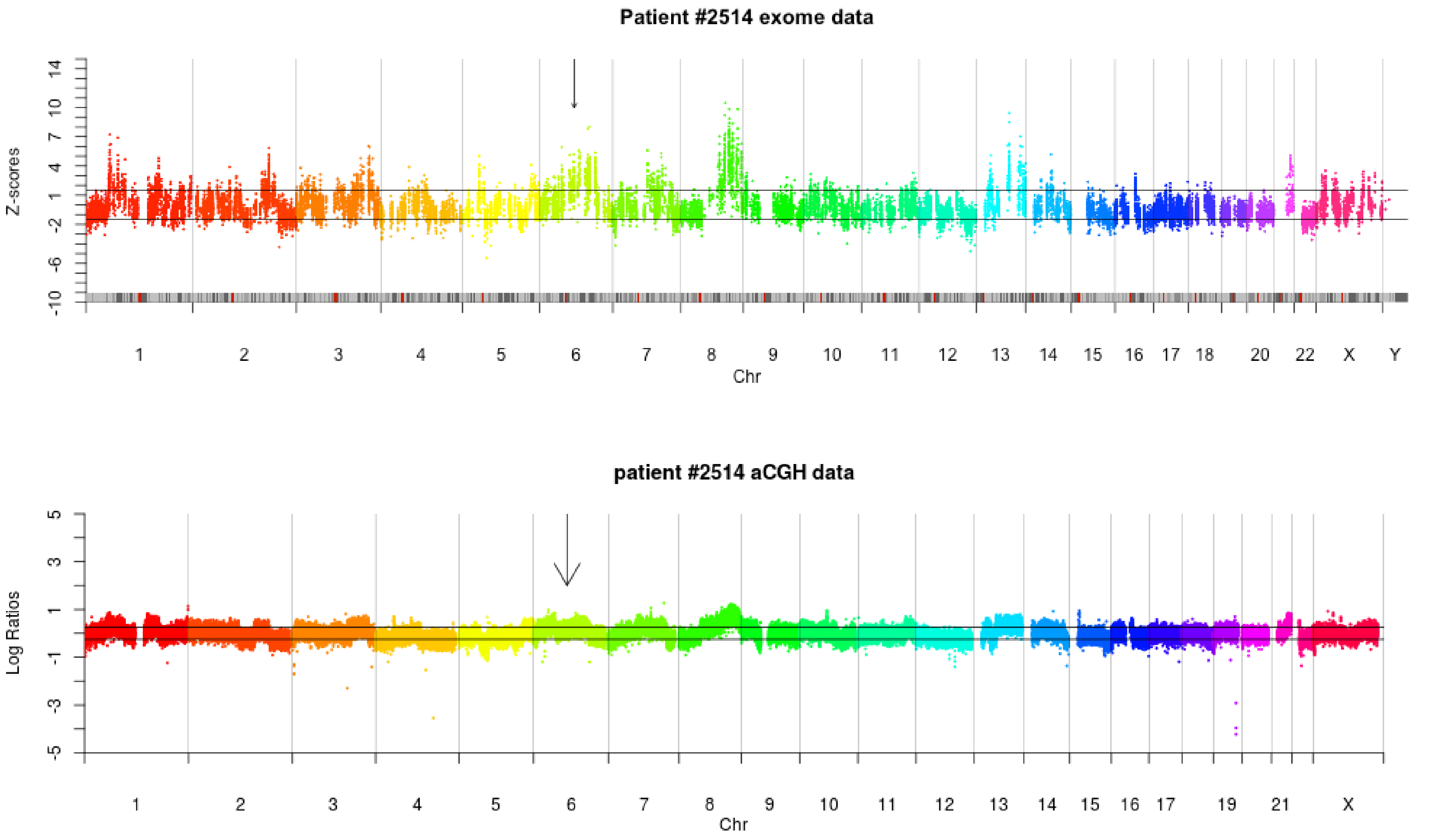
Patient #2514 exome data
Z-scores
14
10
7
4
1
-2
-6
-10
1
2
3
4
5
6
7
8
9
10
11
12
13
14
15
16
17
18
20
22
X
Y
Chr
patient #2514 aCGH data
Log Ratios
5
3
1
-1
-3
-5
1
2
3
4
5
6
7
8
9
10
11
12
13
14
15
16
17
19
21
X
Chr

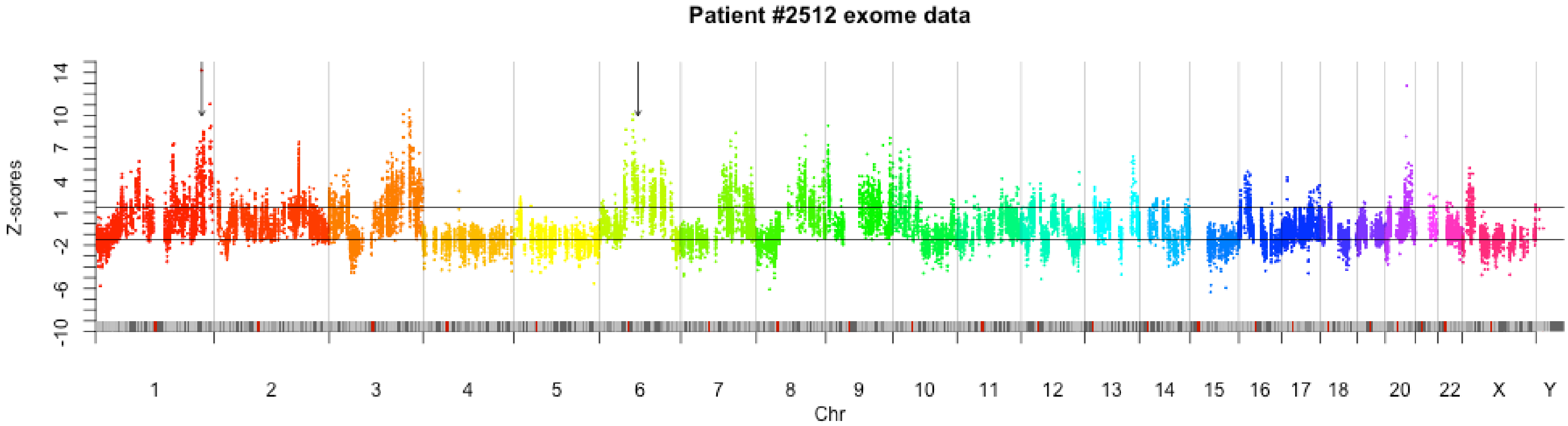
Patient #2512 exome data
Z-scores
14
10
7
4
1
-2
-6
-10
1
2
3
4
5
6
7
8
9
10
11
12
13
14
15
16
17
18
20
22
X
Y
Chr

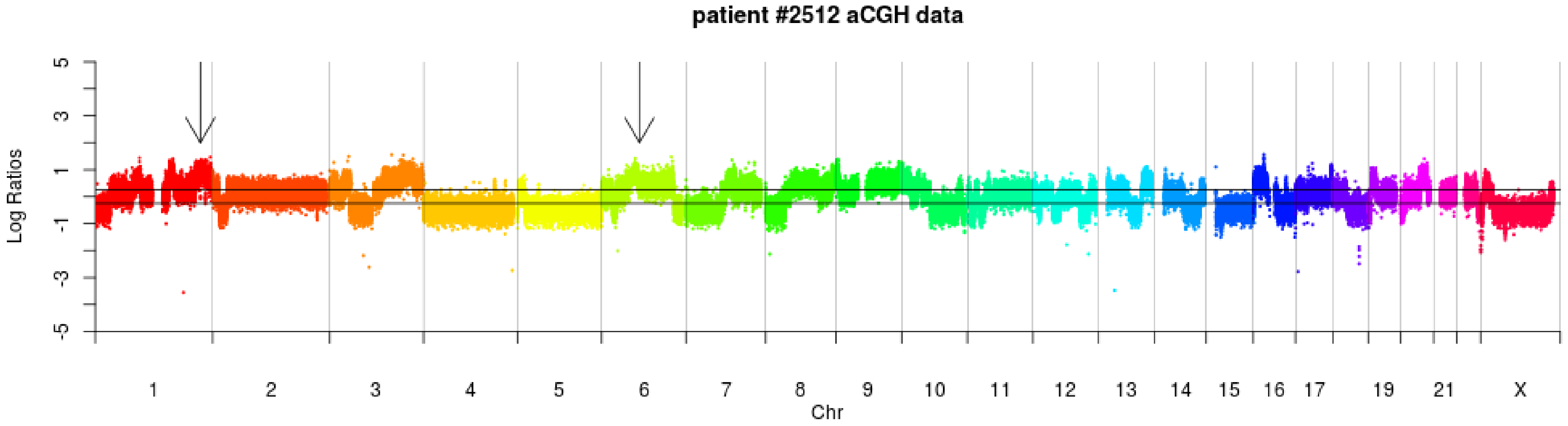
patient #2512 aCGH data
Log Ratios
5
3
1
-1
-3
-5
1
2
3
4
5
6
7
8
9
10
11
12
13
14
15
16
17
19
21
X
Chr

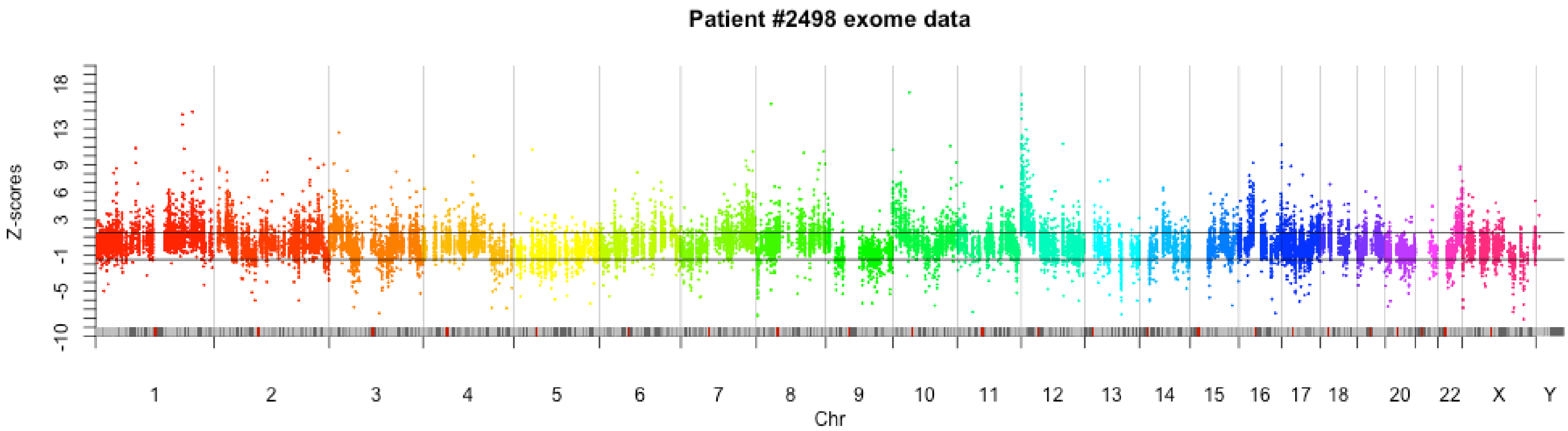
Patient #2498 exome data
Z-scores
18
13
9
6
3
-1
-5
-10
1 2 3 4 5 6 7 8 9 10 11 12 13 14 15 16 17 18 20 22 X Y
Chr

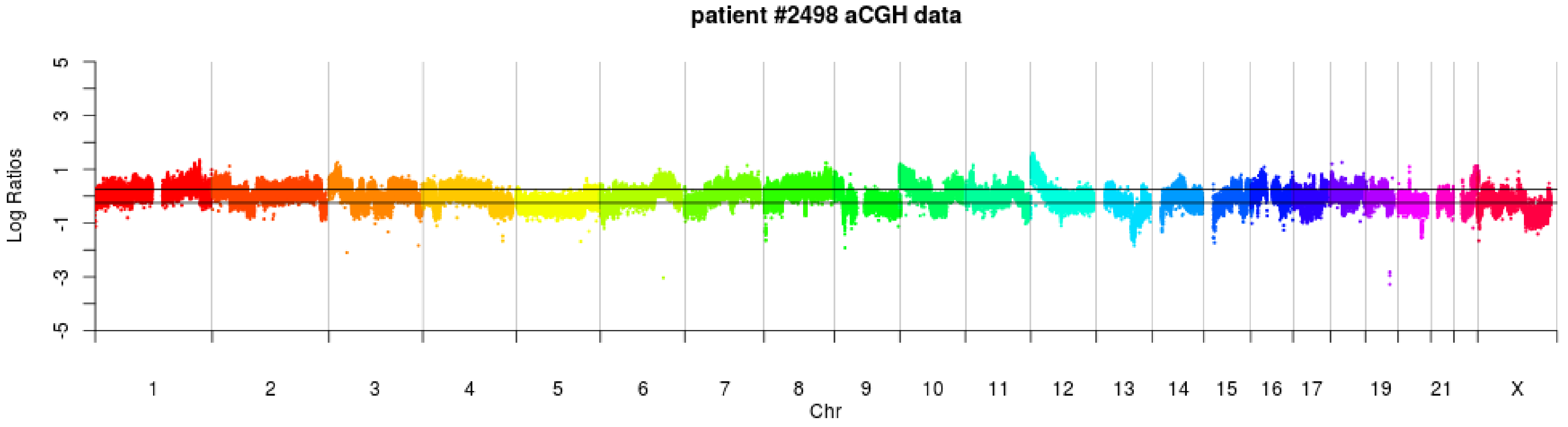
patient #2498 aCGH data
Log Ratios
5
3
1
-1
-3
-5
1 2 3 4 5 6 7 8 9 10 11 12 13 14 15 16 17 19 21 X
Chr

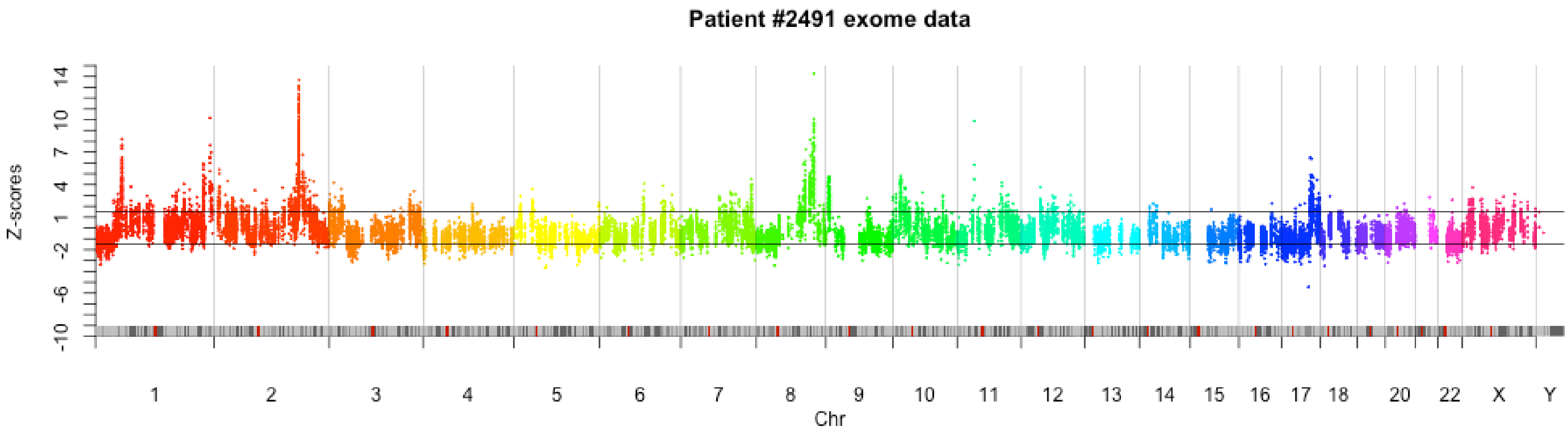
Patient #2491 exome data
Z-scores
14
10
7
4
1
-2
-6
-10
1
2
3
4
5
6
7
8
9
10
11
12
13
14
15
16
17
18
20
22
X
Y
Chr

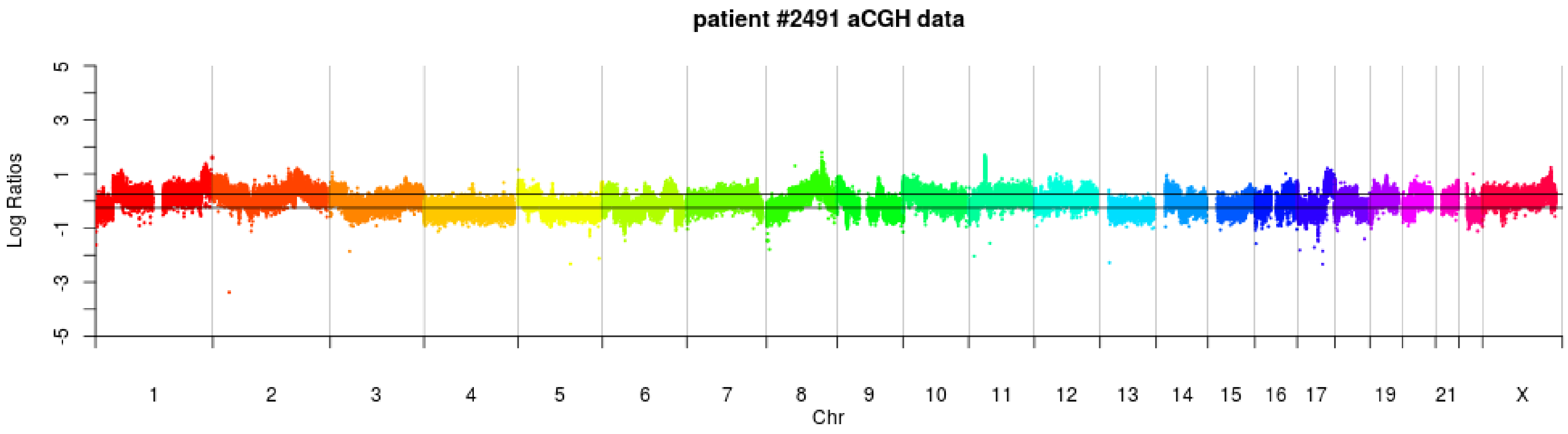
patient #2491 aCGH data
Log Ratios
5
3
1
-1
-3
-5
1
2
3
4
5
6
7
8
9
10
11
12
13
14
15
16
17
19
21
X
Chr

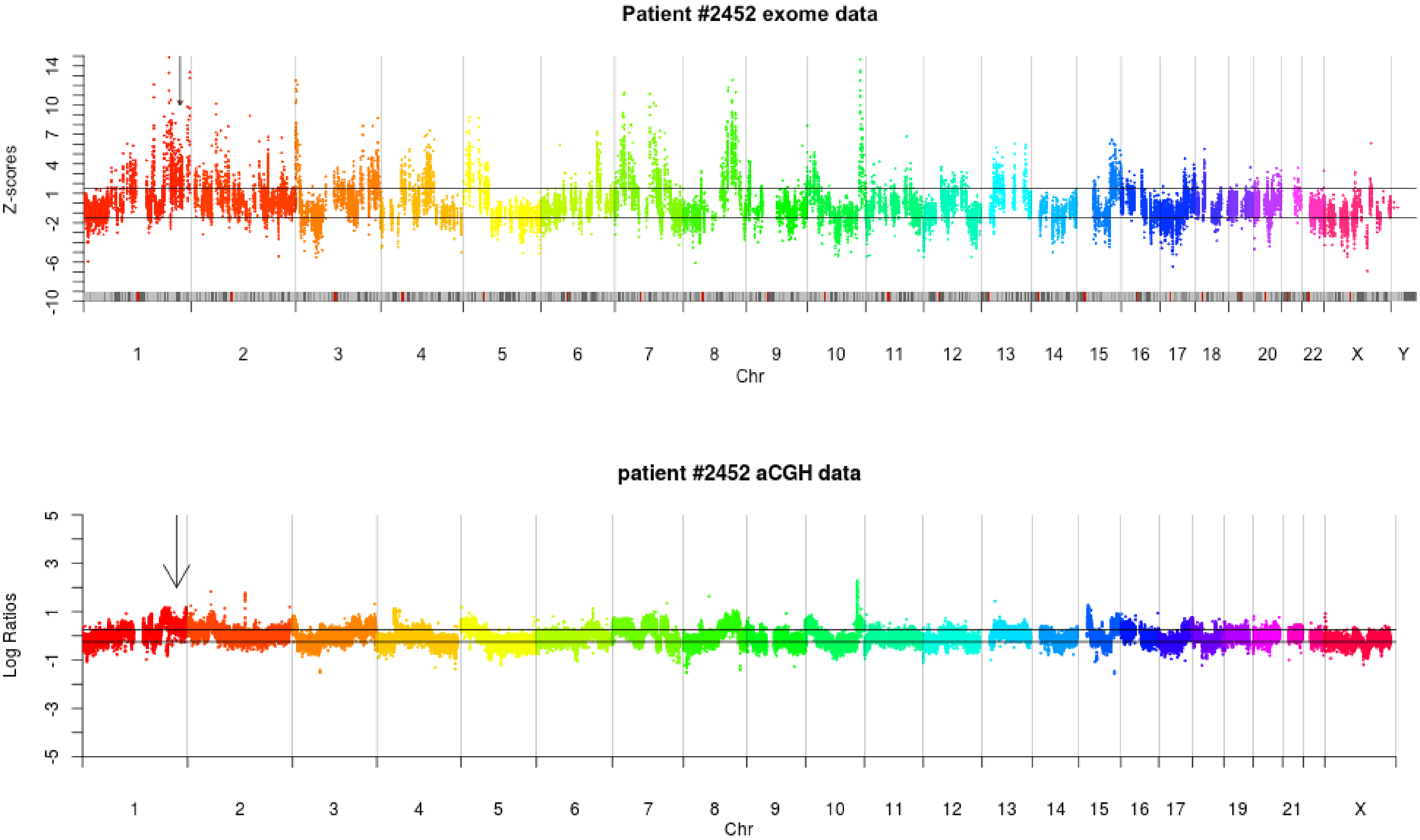

Patient #2452 exome data
Z-scores
14
10
7
4
1
-2
-6
-10
1
2
3
4
5
6
7
8
9
10
11
12
13
14
15
16
17
18
20
22
X
Y
Chr
patient #2452 aCGH data
Log Ratios
5
3
1
-1
-3
-5
1
2
3
4
5
6
7
8
9
10
11
12
13
14
15
16
17
19
21
X
Chr

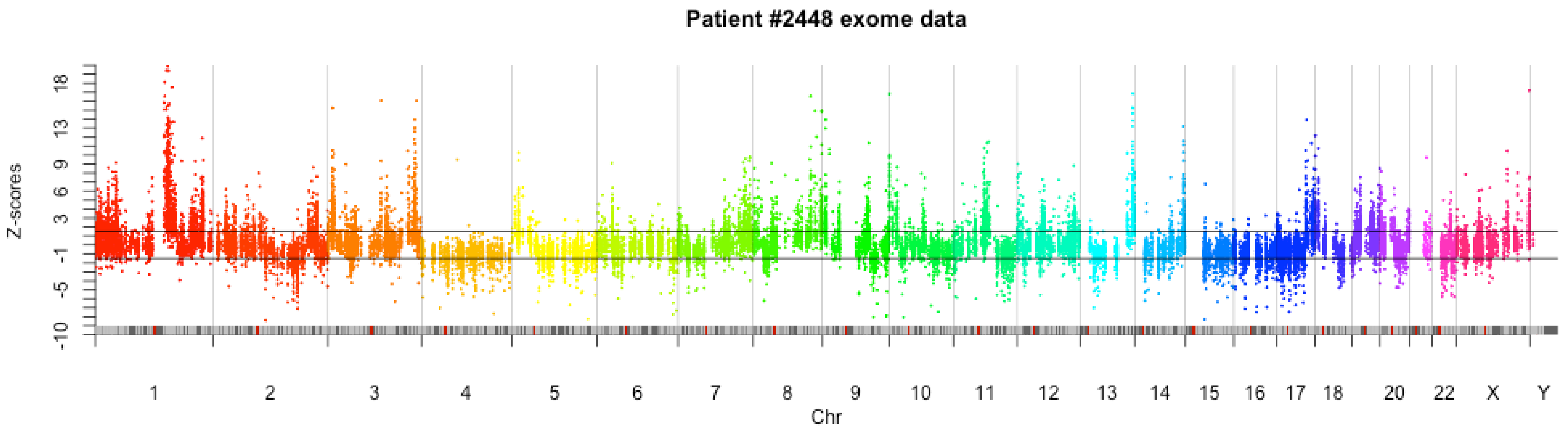
Patient #2448 exome data
Z-scores
18
13
9
6
3
-1
-5
-10
1
2
3
4
5
6
7
8
9
10
11
12
13
14
15
16
17
18
20
22
X
Y
Chr

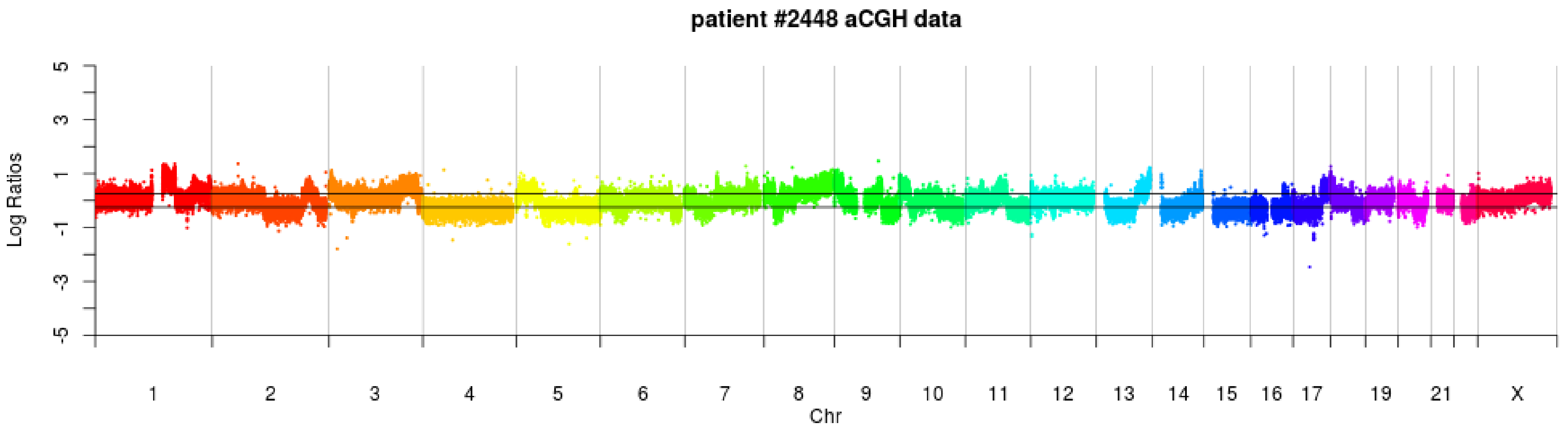
patient #2448 aCGH data
Log Ratios
5
3
1
-1
-3
-5
1
2
3
4
5
6
7
8
9
10
11
12
13
14
15
16
17
19
21
X
Chr

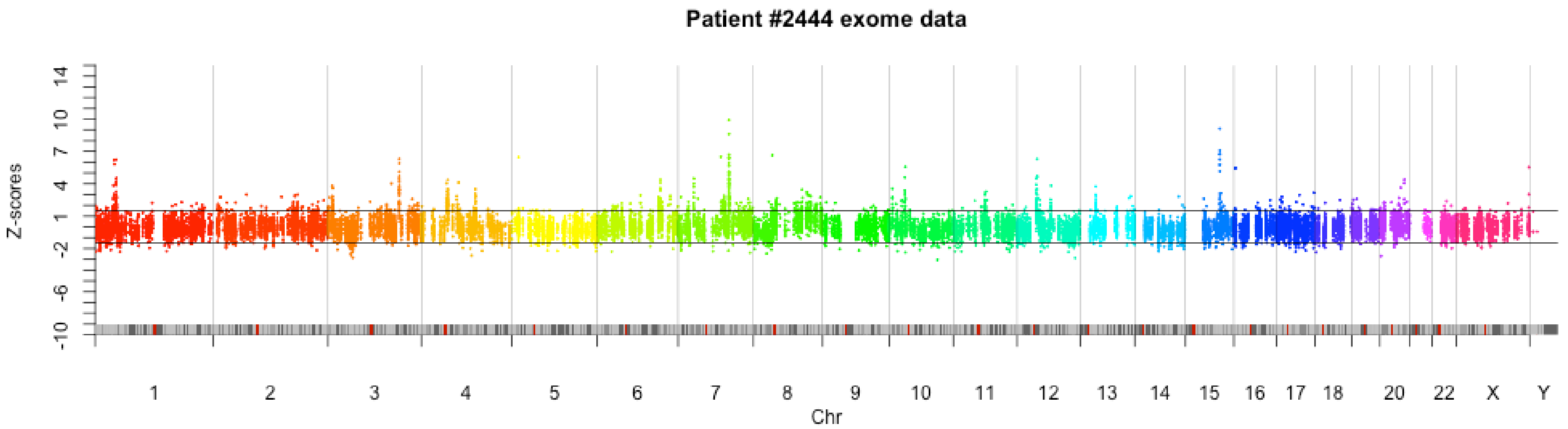

Patient #2444 exome data
Z-scores
14
10
7
4
1
-2
-6
-10
1
2
3
4
5
6
7
8
9
10
11
12
13
14
15
16
17
18
20
22
X
Y
Chr

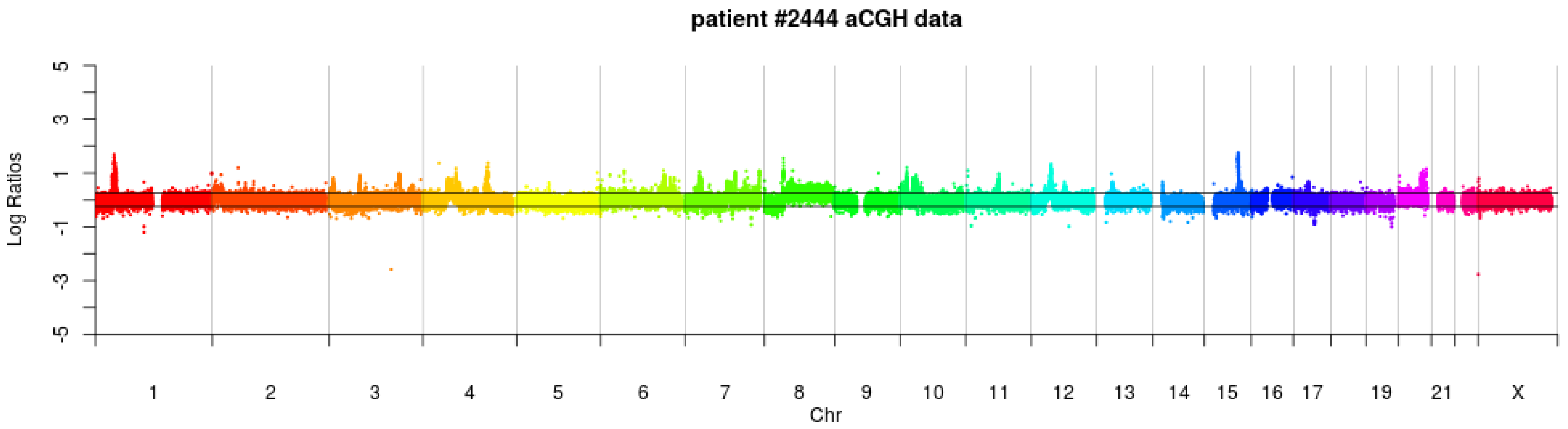

patient #2444 aCGH data
Log Ratios
5
3
1
-1
-3
-5
1
2
3
4
5
6
7
8
9
10
11
12
13
14
15
16
17
19
21
X
Chr

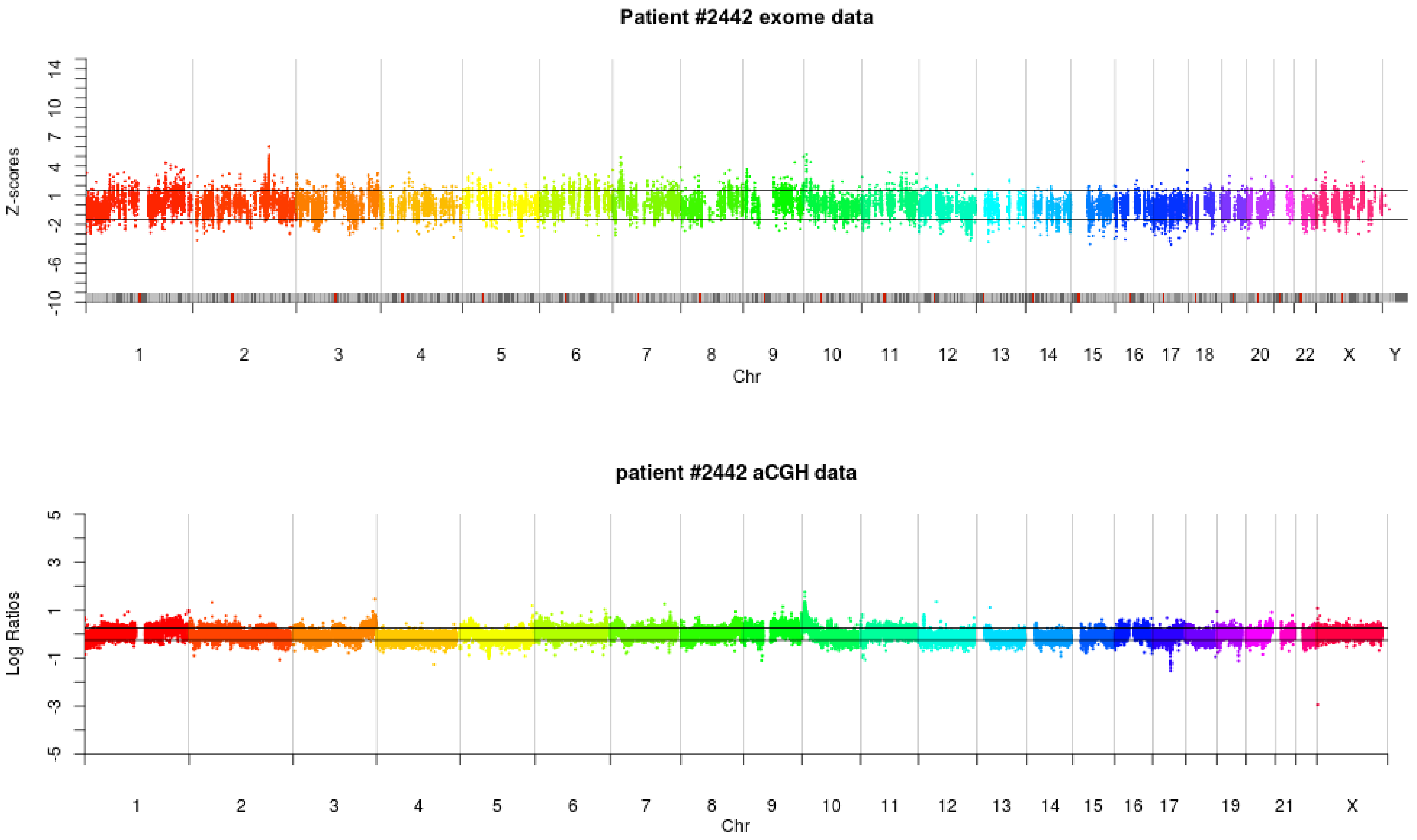
Patient #2442 exome data
Z-scores
14
10
7
4
1
-2
-6
-10
1 2 3 4 5 6 7 8 9 10 11 12 13 14 15 16 17 18 20 22 X Y
Chr
patient #2442 aCGH data
Log Ratios
5
3
1
-1
-3
-5
1 2 3 4 5 6 7 8 9 10 11 12 13 14 15 16 17 19 21 X
Chr

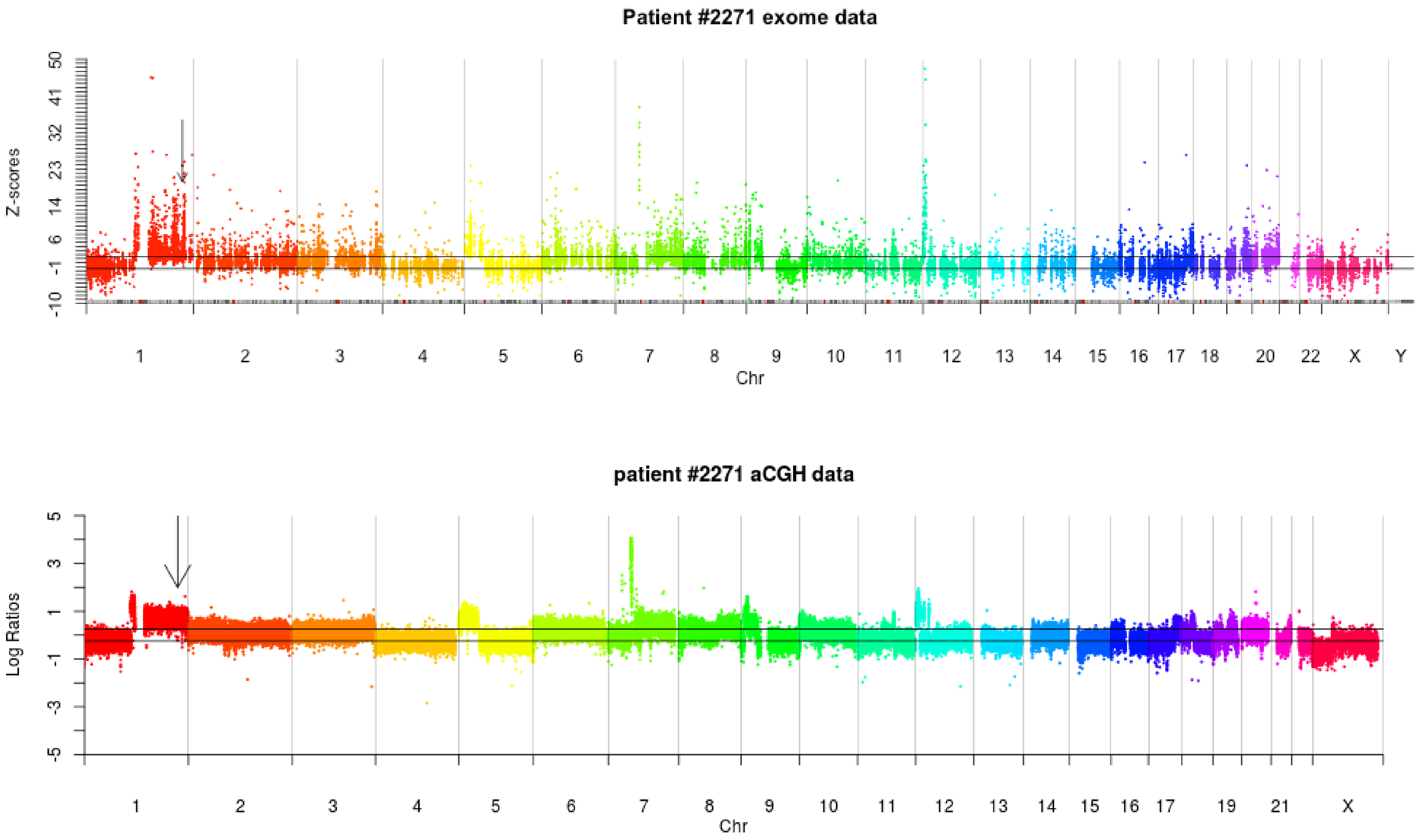
Patient #2271 exome data
Z-scores
50
41
32
23
14
6
-1
-10
1
2
3
4
5
6
7
8
9
10
11
12
13
14
15
16
17
18
20
22
X
Y
Chr
patient #2271 aCGH data
Log Ratios
5
3
1
-1
-3
-5
1
2
3
4
5
6
7
8
9
10
11
12
13
14
15
16
17
19
21
X
Chr

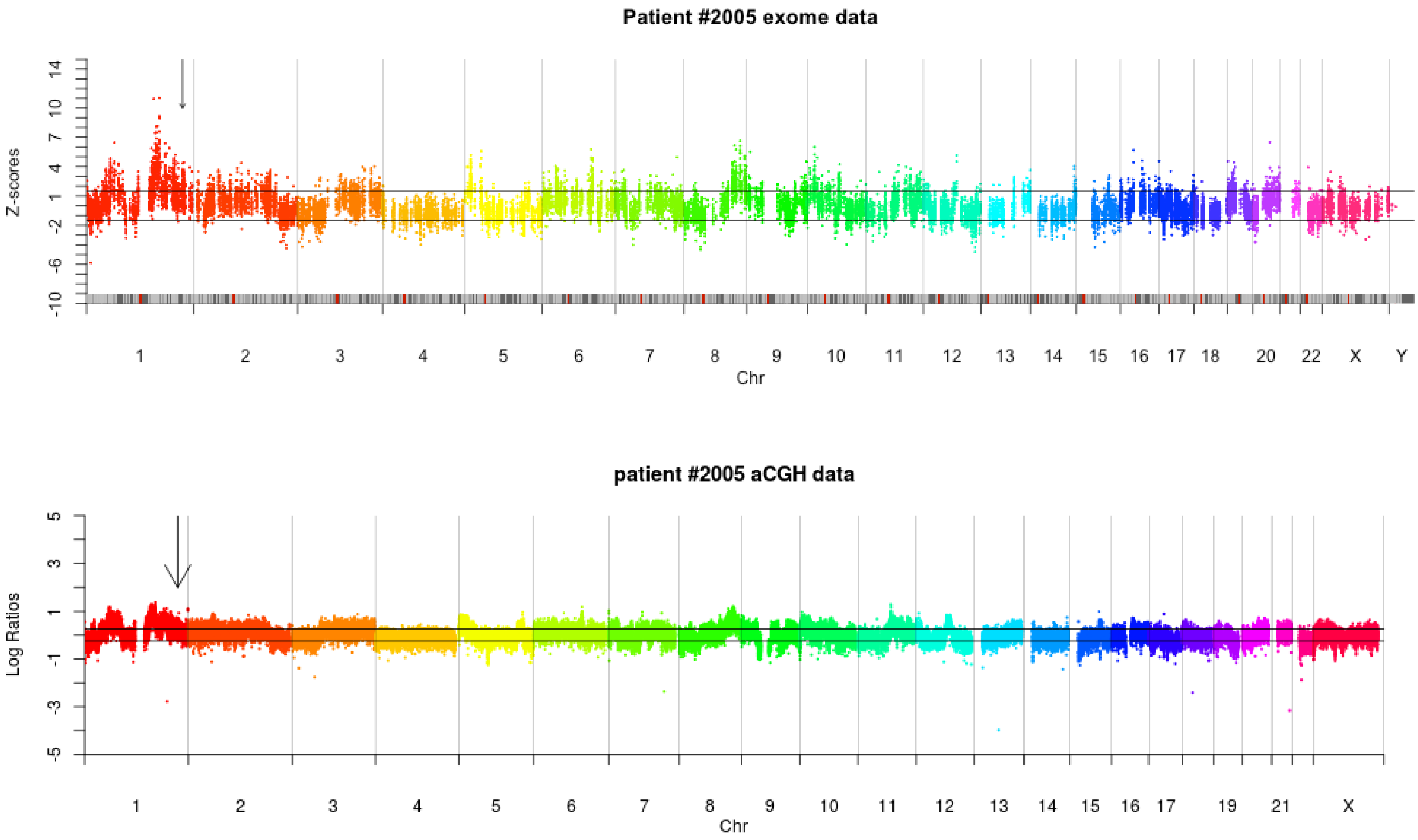
Patient #2005 exome data
Z-scores
Chr
1 2 3 4 5 6 7 8 9 10 11 12 13 14 15 16 17 18 20 22 X Y
patient #2005 aCGH data
Log Ratios
Chr
1 2 3 4 5 6 7 8 9 10 11 12 13 14 15 16 17 19 21 X

Patient #168 exome data

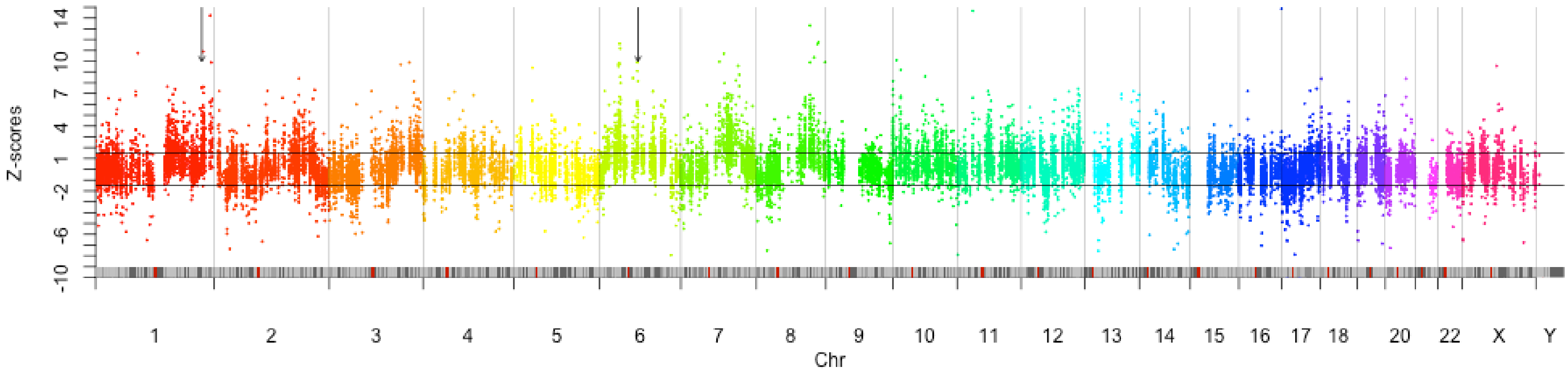

patient #168 aCGH data

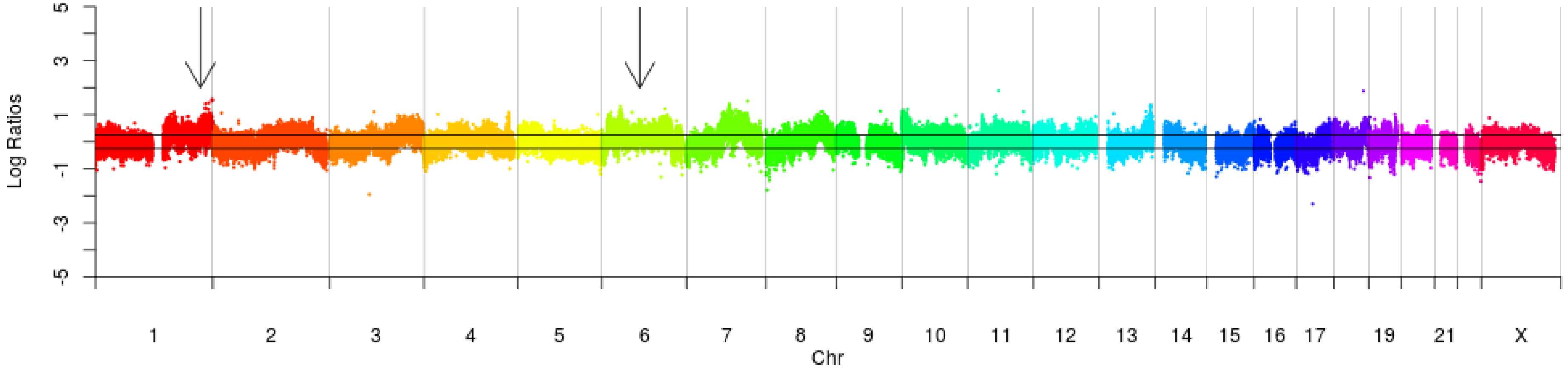

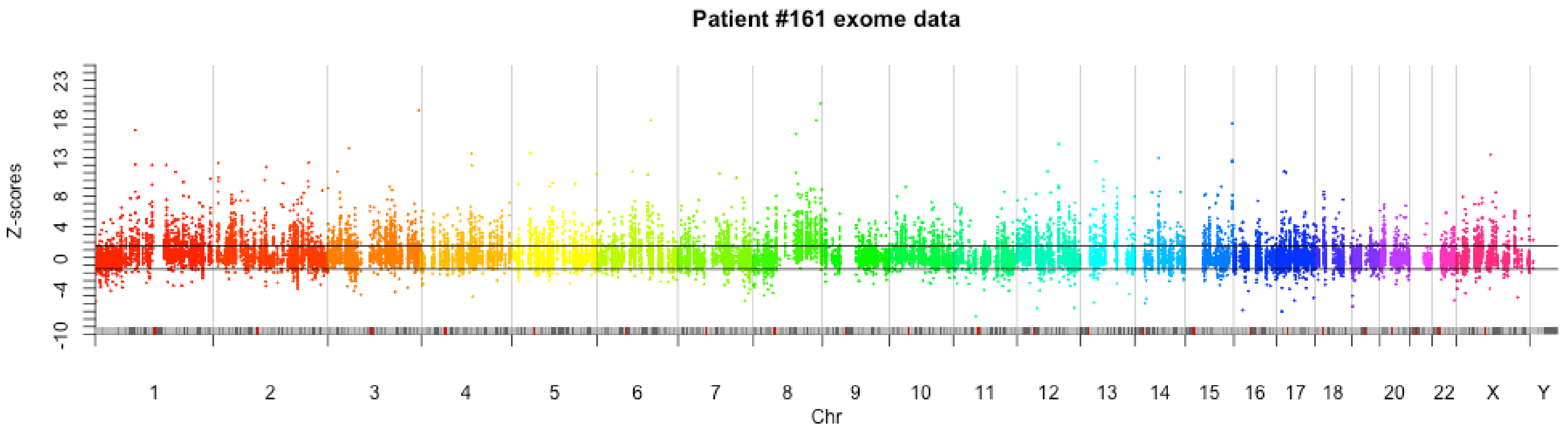
Patient #161 exome data
Z-scores
-10
-4
0
4
8
13
18
23
1
2
3
4
5
6
7
8
9
10
11
12
13
14
15
16
17
18
20
22
X
Y
Chr

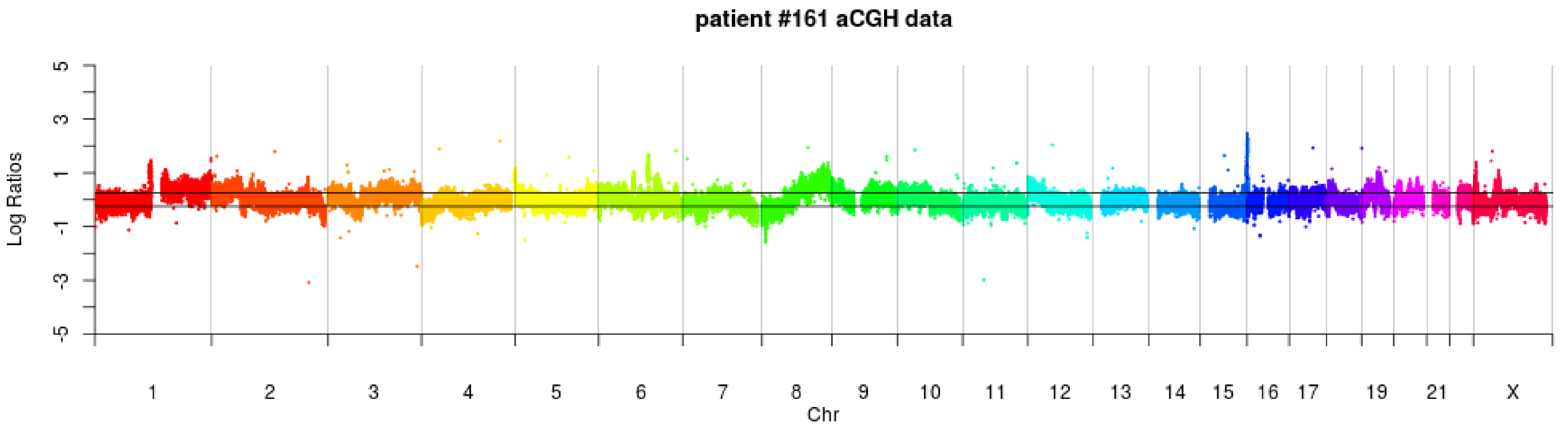
patient #161 aCGH data
Log Ratios
-5
-3
-1
1
3
5
1
2
3
4
5
6
7
8
9
10
11
12
13
14
15
16
17
19
21
X
Chr
