# Supplementary material for: Next generation sequencing of triple negative breast cancer to find predictors for chemotherapy response
Source: Breast Cancer Res. 2015 Oct 3;17:134. doi: 10.1186/s13058-015-0642-8 (PMC4592753; doi:10.1186/s13058-015-0642-8)
Supplement: Additional file 10: Table S7. — Comparison of top alterations listed by Balko et al. (reference [30]) (A) and our study (B) for TCGA [3], Balko et al. [31] and our study. Part A shows the top alterations as listed by Balko (reference [30]) in our study and TCGA (reference [3]), part B shows the top alterations in our study. (DOCX 17 kb) [file 13058_2015_642_MOESM10_ESM.docx]

| **Table S7**  **A.** | |  |  |
| --- | --- | --- | --- |
|  |  |  |  |
|  | This study, Pre-NAC TNBC with alterations (%) (n=56) | Balko et al. Post-NAC TNBC with alterations (%) (n=68-74) | TCGA PAM50 Basal-like with alterations (%) (n=81) |
| TP53 | 55 | 89 | 85 |
| MCL1 | 42 | 54 | 19 |
| MYC | 34 | 35 | 32 |
| PIK3CA | 9 | 12 | 14 |
| PTEN | 8 | 16 | 6 |
| RB1 | 9 | 11 | 11 |
| BRCA1 | 12 | 11 | 12 |
| JAK2 | 11 | 10 | 2 |
| CDKN2A | 8 | 9 | 11 |
| NF1 | 8 | 7 | 2 |
| KRAS | 19 | 7 | 6 |
| CCND1 | 11 | 6 | 2 |
| AKT3 | 31 | 7 | 14 |
| EGFR | 2 | 4 | 2.5 |
| CCND2 | 16 | 6 | 5 |
| CCND3 | 16 | 6 | 2 |
| IGF1R | 9 | 6 | 2 |
| CDK6 | 11 | 6 | 1 |
| CCNE1 | 5 | 6 | 9 |
| **B.** |  |  |  |
| **This study (n=56) (mutations occurring in n>2 samples)** | | |  |
| gene | N | % |  |
| TP53 | 31 | 55% |  |
| TTN | 8 | 14% |  |
| PIK3CA | 5 | 9% |  |
| LRP2 | 4 | 7% |  |
| PTEN | 3 | 5% |  |
| ALMS1 | 3 | 5% |  |
| HSP90AB1 | 3 | 5% |  |
| **This study (n=56) (CNAs), top 10** | |  |  |
| gene | nb.altered | % |  |
| DDR2 | 28 | 44% |  |
| MCL1 | 27 | 42% |  |
| PYGO2 | 24 | 38% |  |
| SCYL3 | 24 | 38% |  |
| EXT1 | 24 | 38% |  |
| TP53BP2 | 23 | 36% |  |
| TIPARP | 23 | 36% |  |
| FZD6 | 23 | 36% |  |
| SNTB1 | 23 | 36% |  |
| TRIB1 | 23 | 36% |  |
